# Supplementary material for: Physical limits to sensing material properties
Source: Nat Commun. 2020 Oct 14;11:5170. doi: 10.1038/s41467-020-18995-4 (PMC7560877; doi:10.1038/s41467-020-18995-4)
Supplement: Supplementary file 2 — Supplementary Information [file 41467_2020_18995_MOESM2_ESM.pdf]

Physical limits to sensing material properties

Beroz et al.

Physical limits to sensing material properties - Supplementary Information

Farzan Beroz,[1,*] Di Zhou,[1] Xiaoming Mao,[1] and David K. Lubensky[1]

[1] *Department of Physics, University of Michigan, Ann Arbor, Michigan 48109, USA*

Supplementary Note 1 The optimal estimator for the Winkler foundation

In this section, we prove that $\hat{\lambda}_0 = s/m$ is the optimal, minimum-variance unbiased estimator (MVUE) of λ_0 for the Winkler foundation. We assume that the sensor has prior knowledge of the model parameters a, ξ, and Δ_λ, as well as of the configurations of the probe fields $f(\boldsymbol{r})$ and $w(\boldsymbol{r})$. We insert Eqs. (5) and (8) into Eq. (7) to find:

$$\hat{\lambda}_0 = \frac{\int w(\boldsymbol{r}) f(\boldsymbol{r}) d\boldsymbol{r}}{\int w(\boldsymbol{r}) u(\boldsymbol{r}) d\boldsymbol{r}}. \qquad \text{(Supplementary Equation 1)}$$

Here, $u(\boldsymbol{r})$ is the response field of the medium in mechanical equilibrium, i.e the solution of:

$$\frac{\delta}{\delta u}(E + \delta E) = \lambda(\boldsymbol{r}) u(\boldsymbol{r}) - f(\boldsymbol{r}) = 0. \qquad \text{(Supplementary Equation 2)}$$

Thus, $u(\boldsymbol{r}) = f(\boldsymbol{r})/\lambda(\boldsymbol{r})$. We take the Taylor expansion of this response field to find:

$$u(\boldsymbol{r}) = \frac{f(\boldsymbol{r})}{\lambda_0}\left(1 - \frac{\delta\lambda(\boldsymbol{r})}{\lambda_0}\right), \qquad \text{(Supplementary Equation 3)}$$

to leading order in $\delta\lambda(\boldsymbol{r})$. Inserting this response field into Supplementary Equation 1 and performing another Taylor expansion yields:

$$\hat{\lambda}_0 = \lambda_0 + \frac{\int \delta\lambda(\boldsymbol{r}) \psi(\boldsymbol{r}) d\boldsymbol{r}}{\int \psi(\boldsymbol{r}) d\boldsymbol{r}}, \qquad \text{(Supplementary Equation 4)}$$

to leading order in $\delta\lambda(\boldsymbol{r})$, where $\psi(\boldsymbol{r}) = f(\boldsymbol{r}) w(\boldsymbol{r})$. To prove that $\hat{\lambda}_0$ is the MVUE for λ_0, we invoke the Lehmann-Scheffé theorem, which states that $\hat{\lambda}_0$ is the MVUE for λ_0 if $\hat{\lambda}_0$ is an (i) unbiased, (ii) sufficient, and (iii) complete statistic for λ_0[1,2]. We consider these conditions in turn:

(i) $\hat{\lambda}_0$ is unbiased.

An unbiased estimator is equal to the estimated quantity on average[3]:

$$\langle \hat{\lambda}_0 \rangle = \lambda_0. \qquad \text{(Supplementary Equation 5)}$$

This equivalence can be shown starting from Supplementary Equation 30 as follows:

$$\langle \hat{\lambda}_0 \rangle = \lambda_0 + \frac{\int \langle \delta\lambda(\boldsymbol{r}) \rangle \psi(\boldsymbol{r}) d\boldsymbol{r}}{\int \psi(\boldsymbol{r}) d\boldsymbol{r}} = \lambda_0, \qquad \text{(Supplementary Equation 6)}$$

where the second equality follows from our definition of $\delta\lambda(\boldsymbol{r})$ as a Gaussian random field with zero mean.

* Corresponding author. Email: farzan@umich.edu

(ii) $\hat{\lambda}_0$ **is sufficient.**

The sufficiency of $\hat{\lambda}_0$ can be established using the Fisher factorization theorem[4]. According to this theorem, a function $\hat{\lambda}_0(\boldsymbol{X})$ of the observed data $\boldsymbol{X}$ is a sufficient statistic for λ_0 if and only if:

$$P(\boldsymbol{X}|\lambda_0) = u(\boldsymbol{X})v(\hat{\lambda}_0(\boldsymbol{X}), \lambda_0), \quad \text{(Supplementary Equation 7)}$$

where u is a nonnegative function that depends only on the data $\boldsymbol{X}$ and v is a nonnegative function that can depend on $\boldsymbol{X}$ as well as λ_0, but for which the only dependence on $\boldsymbol{X}$ is through $\hat{\lambda}_0$.

Here, we take $\boldsymbol{X} = 1/m$. The probability distribution for $\boldsymbol{X}$ conditional on the value of λ_0 is given by:

$$P(\boldsymbol{X}|\lambda_0) = \frac{1}{\sqrt{2\pi\delta\lambda_0^2/s^2}} e^{-\frac{(\boldsymbol{X}-\lambda_0/s)^2}{2\delta\lambda_0^2/s^2}}. \quad \text{(Supplementary Equation 8)}$$

We factor this expression as follows:

$$P(\boldsymbol{X}|\lambda_0) = \left(\frac{1}{\sqrt{2\pi\delta\lambda_0^2/s^2}} e^{-\frac{\boldsymbol{X}^2}{2\delta\lambda_0^2/s^2}}\right)\left(e^{\frac{s\boldsymbol{X}\lambda_0}{\delta\lambda_0^2} - \frac{\lambda_0^2}{2\delta\lambda_0^2}}\right). \quad \text{(Supplementary Equation 9)}$$

We now substitute the estimator $\hat{\lambda}_0 = s\boldsymbol{X}$ into the second exponent to find:

$$P(\boldsymbol{X}|\lambda_0) = \left(\frac{1}{\sqrt{2\pi\delta\lambda_0^2/s^2}} e^{-\frac{\boldsymbol{X}^2}{2\delta\lambda_0^2/s^2}}\right)\left(e^{\frac{\hat{\lambda}_0\lambda_0}{\delta\lambda_0^2} - \frac{\lambda_0^2}{2\delta\lambda_0^2}}\right). \quad \text{(Supplementary Equation 10)}$$

The first and second terms of this expression can be identified, respectively, with the functions u and v in Supplementary Equation 34, which demonstrates the condition (ii) of sufficiency.

(iii) $\hat{\lambda}_0$ **is complete.**

A statistic has the property of completeness if the following relationship holds for every measureable function g[3]:

$$\text{If } \langle g(\hat{\lambda}_0)|\lambda_0\rangle = 0 \text{ for all } \lambda_0, \text{then } P(g(\hat{\lambda}_0) = 0|\lambda_0) = 1 \text{ for all } \lambda_0, \quad \text{(Supplementary Equation 11)}$$

where the conditional average $\langle g(\hat{\lambda}_0)|\lambda_0\rangle$ is given by:

$$\langle g(\hat{\lambda}_0)|\lambda_0\rangle = \frac{1}{\sqrt{2\pi\delta\lambda_0^2}} \int_{-\infty}^{\infty} g(\hat{\lambda}_0) e^{-\frac{(\hat{\lambda}_0-\lambda_0)^2}{2\delta\lambda_0^2}} d\hat{\lambda}_0. \quad \text{(Supplementary Equation 12)}$$

We factor this expression to obtain:

$$\langle g(\hat{\lambda}_0)|\lambda_0\rangle = k(\lambda_0) \int_{-\infty}^{\infty} h(\hat{\lambda}_0) e^{\frac{\hat{\lambda}_0\lambda_0}{\delta\lambda_0^2}} d\hat{\lambda}_0, \quad \text{(Supplementary Equation 13)}$$

where we have defined the functions:

$$k(\lambda_0) = \frac{1}{\sqrt{2\pi\delta\lambda_0^2}} e^{-\frac{\lambda_0^2}{2\delta\lambda_0^2}}, \quad \text{(Supplementary Equation 14)}$$

and

$$h(\hat{\lambda}_0) = g(\hat{\lambda}_0) e^{-\frac{\hat{\lambda}_0^2}{2\delta\lambda_0^2}}. \quad \text{(Supplementary Equation 15)}$$

From Supplementary Equation 40, we see that $\langle g(\hat{\lambda}_0)|\lambda_0\rangle$ is proportional to the two-sided Laplace transform $\mathcal{L}\{h(\hat{\lambda}_0)\}(\lambda_0/\delta\lambda_0^2)$ of $h(\hat{\lambda}_0)$[5]:

$$\langle g(\hat{\lambda}_0)|\lambda_0\rangle = k(\lambda_0)\mathcal{L}\{h(\hat{\lambda}_0)\}(\lambda_0/\delta\lambda_0^2). \quad \text{(Supplementary Equation 16)}$$

To determine whether Supplementary Equation 38 is satisfied, we set this conditional average equal to zero:

$$0 = k(\lambda_0)\mathcal{L}\{h(\hat{\lambda}_0)\}(\lambda_0/\delta\lambda_0^2). \quad \text{(Supplementary Equation 17)}$$

The prefactor $k(\lambda_0)$ is always positive and therefore we can divide both sides by it to find:

$$0 = \mathcal{L}\{h(\hat{\lambda}_0)\}(\lambda_0/\delta\lambda_0^2). \quad \text{(Supplementary Equation 18)}$$

The two-sided Laplace transform is one-to-one[6], and so we must have $h(\hat{\lambda}_0) = 0$ for all values of λ_0. However, since $h(\hat{\lambda}_0)$ is given by $g(\hat{\lambda}_0)$ times a function that is positive for all values of λ_0, we must have $g(\hat{\lambda}_0) = 0$ for all values of λ_0. Thus, the relation Supplementary Equation 38 is satisfied, and $\hat{\lambda}_0$ is a complete statistic for λ_0.

Taken together, the (i) unbiasedness, (ii) sufficiency, and (iii) completeness of $\hat{\lambda}_0$ imply that it is the MVUE of λ_0.

Supplementary Note 2 The optimal estimator for the elastic sheet

In this Supplementary Note, we prove that the optimal, minimum-variance unbiased estimator (MVUE) of λ_0 for the elastic sheet is given by $\hat{\lambda}_0 = s/m$ with $s = \int \psi(\boldsymbol{r})d\boldsymbol{r}$. We start by deriving an expression for m in terms of $\psi(\boldsymbol{r})$. In mechanical equilibrium, the total energy of the medium-sensor system is given by the sum of equations (15) and (4):

$$E + \delta E = \int \left(\frac{1}{2}\lambda(\boldsymbol{r})\nabla u(\boldsymbol{r}) \cdot \nabla u(\boldsymbol{r}) - f(\boldsymbol{r})u(\boldsymbol{r})\right) d\boldsymbol{r}. \quad \text{(Supplementary Equation 19)}$$

We take the variation of this energy with respect to $u(\boldsymbol{r})$ to find the following equation:

$$\nabla \cdot (\lambda(\boldsymbol{r})\nabla u(\boldsymbol{r})) = f(\boldsymbol{r}). \quad \text{(Supplementary Equation 20)}$$

To solve for $u(\boldsymbol{r})$, we invert this equation and perform a Taylor expansion to find:

$$u(\boldsymbol{r}) = \frac{1}{\lambda_0}V_f(\boldsymbol{r}) - \frac{1}{\lambda_0^2}\int G(\boldsymbol{r}-\boldsymbol{r'})\nabla' \cdot (\delta\lambda(\boldsymbol{r'})\nabla' V_f(\boldsymbol{r'}))d\boldsymbol{r'}, \quad \text{(Supplementary Equation 21)}$$

to leading order in $\delta\lambda(\boldsymbol{r})$, where $V_f(\boldsymbol{r})$ is a scalar potential associated with the probe field $f(\boldsymbol{r})$:

$$V_f(\boldsymbol{r}) = \int G(\boldsymbol{r}-\boldsymbol{r'})f(\boldsymbol{r'})d\boldsymbol{r'}, \quad \text{(Supplementary Equation 22)}$$

which is assumed to be continuous and to vanish at infinity. Here, $G(\boldsymbol{r}-\boldsymbol{r'}) = \ln|\boldsymbol{r}-\boldsymbol{r'}|/(2\pi)$ is the response function for a homogeneous sheet, i.e. the solution of $\nabla^2 G(\boldsymbol{r}-\boldsymbol{r'}) = \delta(\boldsymbol{r}-\boldsymbol{r'})$. We insert Supplementary Equation 21 into equation (5) to find:

$$m = \int \left(\frac{w(\boldsymbol{r})V_f(\boldsymbol{r})}{\lambda_0} - \frac{1}{\lambda_0^2}\int w(\boldsymbol{r})G(\boldsymbol{r}-\boldsymbol{r'})\nabla' \cdot (\delta\lambda(\boldsymbol{r'})\nabla' V_f(\boldsymbol{r'}))d\boldsymbol{r'}\right) d\boldsymbol{r}. \quad \text{(Supplementary Equation 23)}$$

To simplify this expression, we define a weight potential $V_w(\boldsymbol{r})$ in analogy with the stimulus potential $V_f(\boldsymbol{r})$:

$$V_w(\boldsymbol{r}) = \int G(\boldsymbol{r}-\boldsymbol{r'})w(\boldsymbol{r'})d\boldsymbol{r'}, \qquad \text{(Supplementary Equation 24)}$$

which we also assume to be continuous and to vanish at infinity. In terms of the probe potentials $V_f(\boldsymbol{r})$ and $V_w(\boldsymbol{r})$, the above expression becomes:

$$m = \int \left(\frac{\nabla^2 V_w(\boldsymbol{r}) V_f(\boldsymbol{r})}{\lambda_0} - \frac{1}{\lambda_0^2} V_w(\boldsymbol{r}) \nabla \cdot (\delta\lambda(\boldsymbol{r}) \nabla V_f(\boldsymbol{r})) \right) d\boldsymbol{r}. \qquad \text{(Supplementary Equation 25)}$$

We integrate both terms by parts to find:

$$m = -\int \left(\frac{1}{\lambda_0} - \frac{\delta\lambda(\boldsymbol{r})}{\lambda_0^2} \right) \psi(\boldsymbol{r}), \qquad \text{(Supplementary Equation 26)}$$

where $\psi(\boldsymbol{r})$ is given by:

$$\psi(\boldsymbol{r}) = \nabla V_f(\boldsymbol{r}) \cdot \nabla V_w(\boldsymbol{r}). \qquad \text{(Supplementary Equation 27)}$$

For simplicity, we have absorbed a minus sign into the definition of $\psi(\boldsymbol{r})$, as a change in sign does not impact the estimator $\hat{\lambda}_0$. Supplementary Equation 26 does not contain boundary terms because we have stipulated that the probe potentials must both vanish at infinity. To cast $\psi(\boldsymbol{r})$ into the form used in equation (16) in the main text, we define the inverse divergence operator $(\nabla\cdot)^{-1}$ such that:

$$(\nabla\cdot)^{-1} f(\boldsymbol{r}) = \nabla V_f(\boldsymbol{r}), \qquad \text{(Supplementary Equation 28)}$$

$$(\nabla\cdot)^{-1} w(\boldsymbol{r}) = \nabla V_w(\boldsymbol{r}). \qquad \text{(Supplementary Equation 29)}$$

Inserting Supplementary Equation 26 into equation (7) and performing another Taylor expansion yields:

$$\hat{\lambda}_0 = \lambda_0 + \frac{\int \delta\lambda(\boldsymbol{r})\psi(\boldsymbol{r})d\boldsymbol{r}}{\int \psi(\boldsymbol{r})d\boldsymbol{r}}, \qquad \text{(Supplementary Equation 30)}$$

to leading order in $\delta\lambda(\boldsymbol{r})$. Inserting the above equation into the definition of the variance $\delta\lambda_0^2$ yields:

$$\delta\lambda_0^2 = \Delta_\lambda \xi^D \frac{\int \psi(\boldsymbol{r})^2 d\boldsymbol{r}}{\left(\int \psi(\boldsymbol{r})d\boldsymbol{r}\right)^2}, \qquad \text{(Supplementary Equation 31)}$$

where we have used equation (3) to perform the average in the definition of the variance. In what follows, we will prove that $\hat{\lambda}_0$ is the MVUE for λ_0, assuming that the sensor has prior knowledge of a, ξ, Δ_λ, $f(\boldsymbol{r})$, and $w(\boldsymbol{r})$. To do so, we invoke the Lehmann-Scheffé theorem, which states that $\hat{\lambda}_0$ is the MVUE for λ_0 if $\hat{\lambda}_0$ is an (*i*) unbiased, (*ii*) sufficient, and (*iii*) complete statistic for λ_0[1,2]. We consider these conditions in turn:

(*i*) $\hat{\lambda}_0$ is unbiased.

An unbiased estimator is equal to the estimated quantity on average[3]:

$$\langle \hat{\lambda}_0 \rangle = \lambda_0. \qquad \text{(Supplementary Equation 32)}$$

This equivalence can be shown starting from Supplementary Equation 30 as follows:

$$\langle \hat{\lambda}_0 \rangle = \lambda_0 + \frac{\int \langle \delta\lambda(\boldsymbol{r}) \rangle \psi(\boldsymbol{r})d\boldsymbol{r}}{\int \psi(\boldsymbol{r})d\boldsymbol{r}} = \lambda_0, \qquad \text{(Supplementary Equation 33)}$$

where the second equality follows from our definition of $\delta\lambda(\boldsymbol{r})$ as a Gaussian random field with zero mean.

(ii) $\hat{\lambda}_0$ **is sufficient.**

The sufficiency of $\hat{\lambda}_0$ can be established using the Fisher factorization theorem[4]. According to this theorem, a function $\hat{\lambda}_0(\boldsymbol{X})$ of the observed data $\boldsymbol{X}$ is a sufficient statistic for λ_0 if and only if:

$$P(\boldsymbol{X}|\lambda_0) = u(\boldsymbol{X})v(\hat{\lambda}_0(\boldsymbol{X}), \lambda_0), \quad \text{(Supplementary Equation 34)}$$

where u is a nonnegative function that depends only on the data $\boldsymbol{X}$ and v is a nonnegative function that can depend on $\boldsymbol{X}$ as well as λ_0, but for which the only dependence on $\boldsymbol{X}$ is through $\hat{\lambda}_0$.

Here, we take $\boldsymbol{X} = 1/m$. The probability distribution for $\boldsymbol{X}$ conditional on the value of λ_0 is given by:

$$P(\boldsymbol{X}|\lambda_0) = \frac{1}{\sqrt{2\pi\delta\lambda_0^2/s^2}} e^{-\frac{(\boldsymbol{X}-\lambda_0/s)^2}{2\delta\lambda_0^2/s^2}}. \quad \text{(Supplementary Equation 35)}$$

We factor this expression as follows:

$$P(\boldsymbol{X}|\lambda_0) = \left(\frac{1}{\sqrt{2\pi\delta\lambda_0^2/s^2}} e^{-\frac{\boldsymbol{X}^2}{2\delta\lambda_0^2/s^2}}\right)\left(e^{\frac{s\boldsymbol{X}\lambda_0}{\delta\lambda_0^2} - \frac{\lambda_0^2}{2\delta\lambda_0^2}}\right). \quad \text{(Supplementary Equation 36)}$$

We now substitute the estimator $\hat{\lambda}_0 = s\boldsymbol{X}$ into the second exponent to find:

$$P(\boldsymbol{X}|\lambda_0) = \left(\frac{1}{\sqrt{2\pi\delta\lambda_0^2/s^2}} e^{-\frac{\boldsymbol{X}^2}{2\delta\lambda_0^2/s^2}}\right)\left(e^{\frac{\hat{\lambda}_0\lambda_0}{\delta\lambda_0^2} - \frac{\lambda_0^2}{2\delta\lambda_0^2}}\right). \quad \text{(Supplementary Equation 37)}$$

The first and second terms of this expression can be identified, respectively, with the functions u and v in Supplementary Equation 34, which demonstrates the condition (ii) of sufficiency.

(iii) $\hat{\lambda}_0$ **is complete.**

A statistic has the property of completeness if the following relationship holds for every measureable function g[3]:

$$\text{If } \langle g(\hat{\lambda}_0)|\lambda_0\rangle = 0 \text{ for all } \lambda_0, \text{ then } P(g(\hat{\lambda}_0) = 0|\lambda_0) = 1 \text{ for all } \lambda_0, \quad \text{(Supplementary Equation 38)}$$

where the conditional average $\langle g(\hat{\lambda}_0)|\lambda_0\rangle$ is given by:

$$\langle g(\hat{\lambda}_0)|\lambda_0\rangle = \frac{1}{\sqrt{2\pi\delta\lambda_0^2}} \int_{-\infty}^{\infty} g(\hat{\lambda}_0) e^{-\frac{(\hat{\lambda}_0-\lambda_0)^2}{2\delta\lambda_0^2}} d\hat{\lambda}_0. \quad \text{(Supplementary Equation 39)}$$

We factor this expression to obtain:

$$\langle g(\hat{\lambda}_0)|\lambda_0\rangle = k(\lambda_0) \int_{-\infty}^{\infty} h(\hat{\lambda}_0) e^{\frac{\hat{\lambda}_0\lambda_0}{\delta\lambda_0^2}} d\hat{\lambda}_0, \quad \text{(Supplementary Equation 40)}$$

where we have defined the functions:

$$k(\lambda_0) = \frac{1}{\sqrt{2\pi\delta\lambda_0^2}} e^{-\frac{\lambda_0^2}{2\delta\lambda_0^2}}, \quad \text{(Supplementary Equation 41)}$$

and

$$h(\hat{\lambda}_0) = g(\hat{\lambda}_0)e^{-\frac{\hat{\lambda}_0^2}{2\delta\lambda_0^2}}. \quad \text{(Supplementary Equation 42)}$$

From Supplementary Equation 40, we see that $\langle g(\hat{\lambda}_0)|\lambda_0\rangle$ is proportional to the two-sided Laplace transform $\mathcal{L}\{h(\hat{\lambda}_0)\}(\lambda_0/\delta\lambda_0^2)$ of $h(\hat{\lambda}_0)$[5]:

$$\langle g(\hat{\lambda}_0)|\lambda_0\rangle = k(\lambda_0)\mathcal{L}\{h(\hat{\lambda}_0)\}(\lambda_0/\delta\lambda_0^2). \quad \text{(Supplementary Equation 43)}$$

To determine whether Supplementary Equation 38 is satisfied, we set this conditional average equal to zero:

$$0 = k(\lambda_0)\mathcal{L}\{h(\hat{\lambda}_0)\}(\lambda_0/\delta\lambda_0^2). \quad \text{(Supplementary Equation 44)}$$

The prefactor $k(\lambda_0)$ is always positive and therefore we can divide both sides by it to find:

$$0 = \mathcal{L}\{h(\hat{\lambda}_0)\}(\lambda_0/\delta\lambda_0^2). \quad \text{(Supplementary Equation 45)}$$

The two-sided Laplace transform is one-to-one[6], and so we must have $h(\hat{\lambda}_0) = 0$ for all values of λ_0. However, since $h(\hat{\lambda}_0)$ is given by $g(\hat{\lambda}_0)$ times a function that is positive for all values of λ_0, we must have $g(\hat{\lambda}_0) = 0$ for all values of λ_0. Thus, Supplementary Equation 38 is satisfied, and $\hat{\lambda}_0$ is a complete statistic for λ_0.

Taken together, the (*i*) unbiasedness, (*ii*) sufficiency, and (*iii*) completeness of $\hat{\lambda}_0$ imply that it is the MVUE of λ_0.

Supplementary Note 3 Probes containing more than three mode pairs break isotropy

In the main text, we showed that incorporating discordant modes into a boundary probe generically yields configurations of $\psi(\boldsymbol{r})$ that vary as a function of the angular coordinate (cf. equation (23)). Here, we demonstrate that although it is possible to cast an isotropic $\psi(\boldsymbol{r})$ using two discordant mode pairs, three or more discordant mode pairs must necessarily break isotropy. To prove this statement, we map the requirement of isotropy onto a constraint satisfaction problem. For simplicity, we will only consider $\psi(\boldsymbol{r})$ in the exterior, which is entirely determined by the values of the probe potentials $V_f(\boldsymbol{r})$ and $V_w(\boldsymbol{r})$ on the boundary of the sensor (where $\nabla^2 V_f(\boldsymbol{r}) = f(\boldsymbol{r})$ and $\nabla^2 V_w(\boldsymbol{r}) = w(\boldsymbol{r})$, see Supplementary Note 1). Here, it is useful to represent a boundary probe in terms of real coefficients $B_k^{(f)}$ and $B_k^{(w)}$ and real phases $\phi_k^{(f)}$ and $\phi_k^{(w)}$ as follows:

$$V_f(\boldsymbol{r}) = \sum_{k=1}^{k_{\max}} B_k^{(f)} \left(\frac{a}{r}\right)^k \cos(k\theta + \phi_k^{(f)}), \quad \text{(Supplementary Equation 46)}$$

$$V_w(\boldsymbol{r}) = \sum_{k=1}^{k_{\max}} B_k^{(w)} \left(\frac{a}{r}\right)^k \cos(k\theta + \phi_k^{(w)}), \quad \text{(Supplementary Equation 47)}$$

In addition, we take each coefficient to be positive. This choice can be made without loss of generality because each term in these expansions is invariant with respect to a change in sign combined with a phase shift of π. Such boundary probes cast the following probe intensity:

$$\psi(\boldsymbol{r}) = \sum_{k,l} B_k^{(f)} B_k^{(w)} k^2 \left(\frac{a}{r}\right)^{2|k|+2} \cos(\phi_k^{(f)} - \phi_k^{(w)}) + B_l^{(f)} B_l^{(w)} l^2 \left(\frac{a}{r}\right)^{2|l|+2} \cos(\phi_l^{(f)} - \phi_l^{(w)})$$
$$+ B_k^{(f)} B_l^{(w)} |k||l| \left(\frac{a}{r}\right)^{|k|+|l|+2} \cos((k-l)\theta + \phi_k^{(f)} - \phi_l^{(w)}) + B_l^{(f)} B_k^{(w)} |k||l| \left(\frac{a}{r}\right)^{|k|+|l|+2} \cos((l-k)\theta + \phi_l^{(f)} - \phi_k^{(w)}).$$
(Supplementary Equation 48)

We see that in order for $\psi(\boldsymbol{r})$ to be independent of the angular coordinate θ, there must be a complete cancellation among the terms arising from the second line. To achieve such a cancellation, the following equation must be satisfied:

$$B_l^{(f)} B_k^{(w)} e^{i(\phi_l^{(f)} - \phi_k^{(w)})} + B_k^{(f)} B_l^{(w)} e^{-i(\phi_k^{(f)} - \phi_l^{(w)})} = 0, \qquad \text{(Supplementary Equation 49)}$$

for all $k \neq l$. In the special case where the probe potentials contain only two mode pairs, these equations can be satisfied if the phases obey:

$$\phi_k^{(f)} + \phi_l^{(f)} = \phi_k^{(w)} + \phi_l^{(w)} + \pi, \qquad \text{(Supplementary Equation 50)}$$

along with the appropriate choice $B_k^{(f)} B_l^{(w)} = B_l^{(f)} B_k^{(w)}$ of amplitudes. These choices result in the following probe intensity:

$$\psi(\boldsymbol{r}) = \frac{B_l^{(f)}}{B_l^{(w)}} \left(B_l^{(w)2} l^2 \left(\frac{r}{a}\right)^{2k} - B_k^{(w)2} k^2 \left(\frac{r}{a}\right)^{2l} \right) \left(\frac{a}{r}\right)^{2+2k+2l} \cos(\phi_2^{(f)} - \phi_2^{(w)}). \qquad \text{(Supplementary Equation 51)}$$

Interestingly, however, for three or more mode pairs, Supplementary Equation 49 cannot be simultaneously satisfied without taking:

$$\phi_k^{(f)} = \phi_k^{(w)} + \frac{\pi}{2}, \qquad \text{(Supplementary Equation 52)}$$

for each mode k. This choice yields $\psi(\boldsymbol{r}) = 0$. Thus, incorporating three or more nonzero mode pairs into a measurement protocol cannot yield a nonzero $\psi(\boldsymbol{r})$ with radial symmetry. This geometrical frustration among modes is analogous to geometrical frustration among spins, which precludes arrangements of three or more spins for which each spin is antiparallel with every other spin.

Supplementary Note 4 The convex relaxation of $\delta\lambda_0/\lambda_0$ for boundary probes

The fractional uncertainty $\delta\lambda_0/\lambda_0$ is a nonconvex function of the coefficients $B_k^{(f)}$ and $B_k^{(w)}$ that appear in equation (24) and equation (26). In this Supplementary Note, we derive an equation for the optimal configurations of $\psi(\boldsymbol{r})$ for the convex relaxation of $\delta\lambda_0/\lambda_0$ presented in the main text. For clarity, we start by representing the probe fields in terms of trigonometric functions as follows:

$$f(\boldsymbol{r}) \sim \delta(r-a) \left(\sum_{k=1}^{k_{\max}} B_k^{(f)} \cos(k\theta) + B_{k+k_{\max}}^{(f)} \sin(k\theta) \right), \qquad \text{(Supplementary Equation 53)}$$

$$w(\boldsymbol{r}) \sim \delta(r-a) \left(\sum_{k=1}^{k_{\max}} B_k^{(w)} \cos(k\theta) + B_{k+k_{\max}}^{(w)} \sin(k\theta) \right), \qquad \text{(Supplementary Equation 54)}$$

instead of the more compact complex representations employed in the main text. Moreover, we will work in units where $a = \Delta_\lambda \xi^D = 1$. In this case, the probe intensity is given by:

$$\psi_\pm(\boldsymbol{r}) = \sum_{k,l=1}^{2k_{\max}} B_{kl} \mathcal{M}_{kl}^{(\pm)}, \qquad \text{(Supplementary Equation 55)}$$

where $B_{kl} = B_k^{(f)} B_l^{(w)}$, and $\mathcal{M}^{(\pm)}$ is a $2k_{\max}$ by $2k_{\max}$ matrix given by:

$$\mathcal{M}^{(\pm)} = \begin{pmatrix} \mathcal{M}^{(1,\pm)} & \mathcal{M}^{(2,\pm)} \\ \mathcal{M}^{(2,\pm)\mathsf{T}} & \mathcal{M}^{(1,\pm)} \end{pmatrix},$$

where

$$\mathcal{M}_{kl}^{(1,\pm)} = klr^{-2\pm k\pm l}\cos((k-l)\theta), \qquad \text{(Supplementary Equation 56)}$$

$$\mathcal{M}_{kl}^{(2,\pm)} = klr^{-2\pm k\pm l}\sin((k-l)\theta). \qquad \text{(Supplementary Equation 57)}$$

The positive and negative versions of these matrices correspond to the interior and the exterior, respectively. For this representation, specifying $B_k^{(f)}$ and $B_k^{(w)}$ is equivalent to specifying an arbitrary rank-one matrix B_{kl} of real coefficients. In this case, we can obtain the convex relaxation defined in the main text by allowing B_{kl} in Supplementary Equation 55 to be an arbitrary real matrix.

Upon performing the convex relaxation, we find that the top and bottom halves of $\mathcal{M}^{(\pm)}$ provide redundant contributions to $\psi_\pm(\boldsymbol{r})$. Thus, we can simplify $\psi_\pm(\boldsymbol{r})$ by consolidating these contributions into two smaller coefficient matrices P_{kl} and Q_{kl} that multiply the entries in the top half of $\mathcal{M}^{(\pm)}$ as follows:

$$\psi_\pm(\boldsymbol{r}) = \sum_{k,l=1}^{k_{\max}} P_{kl}\mathcal{M}_{kl}^{(1,\pm)} + Q_{kl}\mathcal{M}_{kl}^{(2,\pm)}. \qquad \text{(Supplementary Equation 58)}$$

This expression still contains redundant entries, because $\mathcal{M}_{kl}^{(1,\pm)} = \mathcal{M}_{lk}^{(1,\pm)}$ and $\mathcal{M}_{kl}^{(2,\pm)} = -\mathcal{M}_{lk}^{(2,\pm)}$. Thus, without loss of generality, we can simplify the above expression for $\psi_\pm(\boldsymbol{r})$ by taking $P_{kl} = 0$ and $Q_{kl} = 0$ for $l < k$. In addition, a further simplification occurs by noting that $\mathcal{M}_{kk}^{(2,\pm)} = 0$, which implies that $\psi(\boldsymbol{r})$ does not depend on the values of Q_{kk}. Thus, in what follows, we take $Q_{kk} = 0$.

To determine the optimal configurations of $\psi(\boldsymbol{r})$ for this convex relaxation, we will minimize $\delta\lambda_0/\lambda_0$ subject to the constraint $\int \psi(\boldsymbol{r})d\boldsymbol{r} = 1$. We impose this constraint using a Lagrange multiplier γ, which yields the following action S:

$$S = \frac{1}{2}\int_{\mathcal{R}_{\text{int}}}\left(\sum_{k,l=1}^{k_{\max}} P_{kl}\mathcal{M}_{kl}^{(1,+)} + Q_{kl}\mathcal{M}_{kl}^{(2,+)}\right)^2 d\boldsymbol{r} + \frac{1}{2}\int_{\mathcal{R}_{\text{ext}}}\left(\sum_{k,l=1}^{k_{\max}} P_{kl}\mathcal{M}_{kl}^{(1,-)} + Q_{kl}\mathcal{M}_{kl}^{(2,-)}\right)^2 d\boldsymbol{r} + \gamma\sum_{k=1}^{k_{\max}} 2\pi k P_{kk}, \qquad \text{(Supplementary Equation 59)}$$

where the first and second integrals are taken over the interior $\mathcal{R}_{\text{int}}$ and the exterior $\mathcal{R}_{\text{ext}}$ of the sensor, respectively, and the coefficients must satisfy:

$$\sum_{k=1}^{k_{\max}} 2\pi k P_{kk} = 1. \qquad \text{(Supplementary Equation 60)}$$

to ensure $\int \psi(\boldsymbol{r})d\boldsymbol{r} = 1$. By the orthogonality of Supplementary Equation 56 and Supplementary Equation 57, this expression simplifies to:

$$S = \frac{1}{2}\int_{\mathcal{R}_{\text{int}}}\left(\sum_{k,l=1}^{k_{\max}} P_{kl}\mathcal{M}_{kl}^{(1,+)}\right)^2 d\boldsymbol{r} + \frac{1}{2}\int_{\mathcal{R}_{\text{int}}}\left(\sum_{k,l=1}^{k_{\max}} Q_{kl}\mathcal{M}_{kl}^{(2,+)}\right)^2 d\boldsymbol{r} +$$
$$\frac{1}{2}\int_{\mathcal{R}_{\text{ext}}}\left(\sum_{k,l=1}^{k_{\max}} P_{kl}\mathcal{M}_{kl}^{(1,-)}\right)^2 d\boldsymbol{r} + \frac{1}{2}\int_{\mathcal{R}_{\text{ext}}}\left(\sum_{k,l=1}^{k_{\max}} Q_{kl}\mathcal{M}_{kl}^{(2,-)}\right)^2 d\boldsymbol{r} + \gamma\sum_{k=1}^{k_{\max}} 2\pi k P_{kk}. \qquad \text{(Supplementary Equation 61)}$$

To minimize S, we start by separating the sums into diagonal and off-diagonal contributions as follows:

$$S = \frac{1}{2}\int_{\mathcal{R}_{\text{int}}} \left(\sum_{k=1}^{k_{\max}} P_{kk}\mathcal{M}_{kk}^{(1,+)} + \sum_{l>k}^{k_{\max}} P_{kl}\mathcal{M}_{kl}^{(1,+)} \right)^2 d\boldsymbol{r} + \frac{1}{2}\int_{\mathcal{R}_{\text{int}}} \left(\sum_{l>k}^{k_{\max}} Q_{kl}\mathcal{M}_{kl}^{(2,+)} \right)^2 d\boldsymbol{r} +$$
$$\frac{1}{2}\int_{\mathcal{R}_{\text{ext}}} \left(\sum_{k=1}^{k_{\max}} P_{kk}\mathcal{M}_{kk}^{(1,-)} + \sum_{l>k}^{k_{\max}} P_{kl}\mathcal{M}_{kl}^{(1,-)} \right)^2 d\boldsymbol{r} + \frac{1}{2}\int_{\mathcal{R}_{\text{int}}} \left(\sum_{l>k}^{k_{\max}} Q_{kl}\mathcal{M}_{kl}^{(2,-)} \right)^2 d\boldsymbol{r} + \gamma \sum_{k=1}^{k_{\max}} 2\pi k P_{kk}.$$
(Supplementary Equation 62)

These diagonal and off-diagonal contributions are orthogonal to each other, which allows the following simplification:

$$S = \frac{1}{2}\int_{\mathcal{R}_{\text{int}}} \left(\sum_{k=1}^{k_{\max}} P_{kk}\mathcal{M}_{kk}^{(1,+)} \right)^2 d\boldsymbol{r} + \int_{\mathcal{R}_{\text{int}}} \left(\sum_{l>k}^{k_{\max}} P_{kl}\mathcal{M}_{kl}^{(1,+)} \right)^2 d\boldsymbol{r} + \frac{1}{2}\int_{\mathcal{R}_{\text{int}}} \left(\sum_{l>k}^{k_{\max}} Q_{kl}\mathcal{M}_{kl}^{(2,+)} \right)^2 d\boldsymbol{r} +$$
$$\frac{1}{2}\int_{\mathcal{R}_{\text{ext}}} \left(\sum_{k=1}^{k_{\max}} P_{kk}\mathcal{M}_{kk}^{(1,-)} \right)^2 d\boldsymbol{r} + \frac{1}{2}\int_{\mathcal{R}_{\text{ext}}} \left(\sum_{l>k}^{k_{\max}} P_{kl}\mathcal{M}_{kl}^{(1,-)} \right)^2 d\boldsymbol{r} + \frac{1}{2}\int_{\mathcal{R}_{\text{int}}} \left(\sum_{l>k}^{k_{\max}} Q_{kl}\mathcal{M}_{kl}^{(2,-)} \right)^2 d\boldsymbol{r} + \gamma \sum_{k=1}^{k_{\max}} 2\pi k P_{kk}.$$
(Supplementary Equation 63)

From this expression, we see that the off-diagonal contributions can only increase S. Therefore, to minimize S, the coefficients must satisfy $P_{kl} = 0$ and $Q_{kl} = 0$ for $k \neq l$, which correspond to isotropic configurations of $\psi(\boldsymbol{r})$. For these choices, the above equation becomes:

$$S = \frac{1}{2}\int_{\mathcal{R}_{\text{int}}} \left(\sum_{k=1}^{k_{\max}} P_{kk}\mathcal{M}_{kk}^{(1,+)} \right)^2 d\boldsymbol{r} + \frac{1}{2}\int_{\mathcal{R}_{\text{ext}}} \left(\sum_{k=1}^{k_{\max}} P_{kk}\mathcal{M}_{kk}^{(1,-)} \right)^2 d\boldsymbol{r} + \gamma \sum_{k=1}^{k_{\max}} 2\pi k P_{kk}.$$ (Supplementary Equation 64)

To minimize S, the remaining undetermined coefficients P_{kk} must satisfy $dS/dP_{kk} = 0$, which corresponds to the following equation:

$$\int_{\mathcal{R}_{\text{int}}} \left(\mathcal{M}_{mn}^{(1,+)} \sum_{k=1}^{k_{\max}} P_{kk}\mathcal{M}_{kk}^{(1,+)} \right) d\boldsymbol{r} + \int_{\mathcal{R}_{\text{ext}}} \left(\mathcal{M}_{mn}^{(1,-)} \sum_{k=1}^{k_{\max}} P_{kk}\mathcal{M}_{kk}^{(1,-)} \right) d\boldsymbol{r} = -2\pi\gamma m \delta_{m,n},$$
(Supplementary Equation 65)

where $\mathcal{M}_{kk}^{(1,\pm)} = k^2 r^{-2\pm 2k}$. To determine the coefficients P_{kk}, we take $m = n$ in the above equation and perform the integrals to find:

$$\sum_{k=1}^{k_{\max}} P_{kk} C_{km} = -\gamma m,$$ (Supplementary Equation 66)

where C_{km} is a matrix given by:

$$C_{km} = \frac{1}{2}\left(\frac{k^2 m^2}{k+m-1} + \frac{k^2 m^2}{k+m+1} \right).$$ (Supplementary Equation 67)

Thus, the coefficients P_{kk} are given by:

$$P_{kk} = -\gamma \sum_{m=1}^{k_{\max}} m C_{km}^{-1},$$ (Supplementary Equation 68)

where γ can be determined by inserting the above equation into Supplementary Equation 60, which yields:

$$\gamma = -\left(2\pi \sum_{k,m=1}^{k_{\max}} kmC_{km}^{-1}\right)^{-1}. \quad \text{(Supplementary Equation 69)}$$

To explore the behavior of $\delta\lambda_0/\lambda_0$ for large $k_{\max}$, we determined the coefficients P_{kk} by numerically inverting C_{km} and solving Supplementary Equation 68 and Supplementary Equation 69. We then inserted the resulting $\psi(\boldsymbol{r})$ into Supplementary Equation 31. This calculation yields values of $\delta\lambda_0/\lambda_0$ that rapidly converge to $\delta\lambda_{0,\text{low}}/\lambda_0 \approx 1/\sqrt{\pi}$ as $k_{\max}$ is increased. This convergence suggests a representation of π that, to our knowledge, has not previously been reported in the literature. A simplified form of this representation can be obtained by rescaling the matrix $C_{km} \to \tilde{C}_{km}$, where:

$$\tilde{C}_{km} = \frac{km}{k+m-1} + \frac{km}{k+m+1}. \quad \text{(Supplementary Equation 70)}$$

In terms of this matrix, our results suggest the following identity:

$$\lim_{N\to\infty} \sum_{k,m=1}^{N} \tilde{C}_{km}^{-1} = \frac{\pi}{4}. \quad \text{(Supplementary Equation 71)}$$

Supplementary Note 5 The optimal estimator for multiple probes

A sensor that performs multiple probes can make a more precise estimate of λ_0. To do so, the sensor must take advantage of the correlations among the probes by combining them with appropriately chosen weights, such that the deviations of different measurements away from λ_0 cancel each other out. In this Supplementary Note, we prove that the minimum-variance unbiased estimator (MVUE) of λ_0 for a sequence of probes is given by the best linear unbiased sum of the estimators for individual probes. We then represent the MVUE in terms of an effective probe intensity and derive an equation for its variance.

(i) unbiased estimator of λ_0

The most general linear estimator is given by:

$$\hat{\lambda}_0 = \sum_k q_k \hat{\lambda}_{0,k}, \quad \text{(Supplementary Equation 72)}$$

where q_k are constant weights assigned to each measurement protocol k. For $\hat{\lambda}_0$ to be unbiased, we must have $\langle\hat{\lambda}_0\rangle = \lambda_0$, which implies:

$$\sum_k q_k = 1. \quad \text{(Supplementary Equation 73)}$$

The variance $\delta\lambda_0^2$ of the estimator is given by:

$$\delta\lambda_0^2 = \sum_{k,l} C_{kl} q_k q_l, \quad \text{(Supplementary Equation 74)}$$

where $C_{kl} = \langle(\hat{\lambda}_{0,k} - \lambda_0)(\hat{\lambda}_{0,l} - \lambda_0)\rangle$ is the covariance matrix of the estimators for the individual probes k. To determine the best linear estimator, we minimize this variance with respect to the weights q_k, subject to the constraint Supplementary Equation 73. The optimal q_k must satisfy the following equation:

$$\frac{d}{dq_k}\left(\sum_{k,l} C_{kl} q_k q_l - 2\gamma \sum_k q_k\right) = 0, \qquad \text{(Supplementary Equation 75)}$$

where 2γ is a Lagrange multiplier that enforces Supplementary Equation 73, and we have included the factor of 2 for later convenience. The above equation simplifies to yield the following equation for q_k:

$$\sum_l C_{kl} q_l = \gamma. \qquad \text{(Supplementary Equation 76)}$$

Thus, the optimal weights are given by:

$$q_k = \gamma \sum_l C^{-1}_{kl}. \qquad \text{(Supplementary Equation 77)}$$

To solve for γ, we sum over the index k and apply the unbiasedness constraint Supplementary Equation 73 to find:

$$\gamma = \left(\sum_{k,l} C^{-1}_{kl}\right)^{-1}. \qquad \text{(Supplementary Equation 78)}$$

Thus, the best unbiased linear estimator $\hat{\lambda}_0$ is given by:

$$\hat{\lambda}_0 = \frac{\sum_{k,l} C^{-1}_{kl} \hat{\lambda}_{0,k}}{\sum_{k,l} C^{-1}_{kl}}. \qquad \text{(Supplementary Equation 79)}$$

By the Lehmann-Scheffé theorem[1,2], $\hat{\lambda}_0$ is the MVUE for λ_0 if $\hat{\lambda}_0$ is an (*i*) unbiased, (*ii*) sufficient, and (*iii*) complete statistic for λ_0. It remains to be shown that (*ii*) and (*iii*) are satisfied.

(*ii*) $\hat{\lambda}_0$ is sufficient.

According to the Fisher factorization theorem[4], $\hat{\lambda}_0(\hat{\lambda}_{0,k})$ with $\hat{\lambda}_{0,k}$ drawn from the conditional probability distribution $P(\hat{\lambda}_{0,k}|\lambda_0)$ is a sufficient statistic for λ_0 if:

$$P(\hat{\lambda}_{0,k}|\lambda_0) = u(\hat{\lambda}_{0,k}) v(\hat{\lambda}_0(\hat{\lambda}_{0,k}), \lambda_0), \qquad \text{(Supplementary Equation 80)}$$

where u is a function that depends only on the individual estimators $\hat{\lambda}_{0,k}$ and v is a function that can depend on $\hat{\lambda}_{0,k}$ as well as λ_0, but for which the only dependence on $\hat{\lambda}_{0,k}$ is through $\hat{\lambda}_0$. The probability distribution for $\hat{\lambda}_{0,k}$ is given by the following multivariate normal distribution:

$$P(\hat{\lambda}_{0,k}|\lambda_0) = \frac{1}{\sqrt{(2\pi)^N |C_{kl}|}} e^{-\frac{1}{2}\sum_{k,l}(\hat{\lambda}_{0,k}-\lambda_0) C^{-1}_{kl} (\hat{\lambda}_{0,k}-\lambda_0)}, \qquad \text{(Supplementary Equation 81)}$$

where N is the total number of probes and $|C_{kl}|$ is the determinant of C_{kl}. We perform the Fisher factorization by first expanding the exponent as follows:

$$P(\hat{\lambda}_{0,k}|\lambda_0) = \frac{1}{\sqrt{(2\pi)^N |C_{kl}|}} e^{-\frac{1}{2}\sum_{k,l}\left(\hat{\lambda}_{0,k} C^{-1}_{kl} \hat{\lambda}_{0,l} - 2\lambda_0 C^{-1}_{kl} \hat{\lambda}_{0,l} + \lambda_0^2 C^{-1}_{kl}\right)}. \qquad \text{(Supplementary Equation 82)}$$

We now insert Supplementary Equation 78 and Supplementary Equation 79 into the second and third terms of the exponent and factor to find:

$$P(\hat{\lambda}_{0,k}|\lambda_0) = \left(\frac{1}{\sqrt{(2\pi)^N |C_{kl}|}} e^{-\frac{1}{2}\sum_{k,l}\hat{\lambda}_{0,k} C_{kl}^{-1} \hat{\lambda}_{0,l}} \right) \left(e^{\lambda_0 \gamma \hat{\lambda}_0 - \frac{1}{2}\lambda_0^2 \gamma} \right). \quad \text{(Supplementary Equation 83)}$$

The first and second terms of this expression can be identified, respectively, with the functions u and v in Supplementary Equation 80, which demonstrates the condition (*ii*) of sufficiency.

(iii) $\hat{\lambda}_0$ is complete.

A statistic has the property of completeness if the following relationship holds for every measureable function g[3]:

$$\text{If } \langle g(\hat{\lambda}_0)|\lambda_0\rangle = 0 \text{ for all } \lambda_0, \text{ then } P(g(\hat{\lambda}_0)|\lambda_0) = 1 \text{ for all } \lambda_0, \quad \text{(Supplementary Equation 84)}$$

where the conditional average $\langle g(\hat{\lambda}_0)|\lambda_0\rangle$ is given by:

$$\langle g(\hat{\lambda}_0)|\lambda_0\rangle = \frac{1}{\sqrt{(2\pi)^N |C_{kl}|}} \int_{-\infty}^{\infty} g(\hat{\lambda}_0) e^{-\frac{1}{2}\sum_{k,l}(\hat{\lambda}_{0,k}-\lambda_0) C_{kl}^{-1} (\hat{\lambda}_{0,k}-\lambda_0)} \prod_k d\hat{\lambda}_{0,k}. \quad \text{(Supplementary Equation 85)}$$

We factor this expression to obtain:

$$\langle g(\hat{\lambda}_0)|\lambda_0\rangle = t(\lambda_0) \int_{-\infty}^{\infty} h(\{\hat{\lambda}_{0,k}\}) e^{\hat{\lambda}_0 \gamma \lambda_0} \prod_k d\hat{\lambda}_{0,k}, \quad \text{(Supplementary Equation 86)}$$

where we have defined the functions:

$$t(\lambda_0) = \frac{1}{\sqrt{(2\pi)^N |C_{kl}|}} e^{-\frac{\gamma \lambda_0^2}{2}}, \quad \text{(Supplementary Equation 87)}$$

and

$$h(\{\hat{\lambda}_{0,k}\}) = g(\hat{\lambda}_0) e^{-\frac{1}{2}\sum_{k,l}\hat{\lambda}_{0,k} C_{kl}^{-1} \hat{\lambda}_{0,l}}. \quad \text{(Supplementary Equation 88)}$$

From Supplementary Equation 86, we see that $\langle g(\hat{\lambda}_0)|\lambda_0\rangle$ is proportional to the two-sided multivariate Laplace transform $\mathcal{L}\{h(\{\hat{\lambda}_{0,k}\})\}(\gamma\lambda_0)$ of $h(\{\hat{\lambda}_{0,k}\})$[5]:

$$\langle g(\hat{\lambda}_0)|\lambda_0\rangle = t(\lambda_0) \mathcal{L}\{h(\{\hat{\lambda}_{0,k}\})\}(\gamma\lambda_0). \quad \text{(Supplementary Equation 89)}$$

To determine whether Supplementary Equation 84 is satisfied, we now assume that this conditional average is equal to zero:

$$0 = t(\lambda_0) \mathcal{L}\{h(\{\hat{\lambda}_{0,k}\})\}(\gamma\lambda_0). \quad \text{(Supplementary Equation 90)}$$

The prefactor $t(\lambda_0)$ is always positive and therefore we can divide both sides by it to find:

$$0 = \mathcal{L}\{h(\{\hat{\lambda}_{0,k}\})\}(\gamma\lambda_0). \quad \text{(Supplementary Equation 91)}$$

The two-sided Laplace transform is one-to-one[6], and so we must have $h(\{\hat{\lambda}_{0,k}\}) = 0$ for all values of λ_0. However, since $h(\{\hat{\lambda}_{0,k}\})$ is given by $g(\hat{\lambda}_0)$ times a function that is positive for all values of λ_0, we must have $g(\hat{\lambda}_0) = 0$ for all values of λ_0. Thus, Supplementary Equation 84 is satisfied, and $\hat{\lambda}_0$ is a complete statistic for λ_0.

Taken together, the (*i*) unbiasedness, (*ii*) sufficiency, and (*iii*) completeness of $\hat{\lambda}_0$ imply that it is the MVUE for λ_0.

(iv) $\hat{\lambda}_0$ in terms of $\Psi(\boldsymbol{r})$

In the main text, we represented $\hat{\lambda}_0$ in terms of an effective probe intensity $\Psi(\boldsymbol{r})$. Here, we demonstrate that this representation is equivalent to Supplementary Equation 79 for $\hat{\lambda}_0$ derived above. To do so, we insert equation (29) into equation (28) to find:

$$\hat{\lambda}_0 = \lambda_0 + \frac{\int \sum_{k,l} \left(C^{-1}_{kl} \frac{\psi_k(\boldsymbol{r})}{\int \psi_k(\boldsymbol{r}) d\boldsymbol{r}} \right) \delta\lambda(\boldsymbol{r}) d\boldsymbol{r}}{\sum_{k,l} C^{-1}_{kl}}. \qquad \text{(Supplementary Equation 92)}$$

We then use equation (9) for the estimator of an individual probe to simplify this expression, which yields Supplementary Equation 79. The coefficients p_k that appear in equation (29) in the main text are related to the coefficients q_k above by $q_k = p_k \gamma = p_k / (\sum_{k,l} C^{-1}_{kl})$.

(v) Variance of $\hat{\lambda}_0$

The variance $\delta\lambda_0^2$ of the estimator is defined by:

$$\delta\lambda_0^2 = \langle (\hat{\lambda}_0 - \lambda_0)^2 \rangle. \qquad \text{(Supplementary Equation 93)}$$

We insert Supplementary Equation 79 into the above expression to find:

$$\delta\lambda_0^2 = \left\langle \left(\frac{\sum_{k_1,l_1} C^{-1}_{k_1 l_1} (\hat{\lambda}_{0,k_1} - \lambda_0)}{\sum_{k_1,l_1} C^{-1}_{k_1 l_1}} \right) \left(\frac{\sum_{k_2,l_2} C^{-1}_{k_2 l_2} (\hat{\lambda}_{0,k_2} - \lambda_0)}{\sum_{k_2,l_2} C^{-1}_{k_2 l_2}} \right) \right\rangle. \qquad \text{(Supplementary Equation 94)}$$

This expression can be simplified by factoring the denominators and invoking the definition of the covariance matrix:

$$\delta\lambda_0^2 = \frac{1}{\left(\sum_{k,l} C^{-1}_{kl} \right)^2} \sum_{k_1,l_1} \sum_{k_2,l_2} C^{-1}_{k_1 l_1} C^{-1}_{k_2 l_2} C_{k_1 l_2}. \qquad \text{(Supplementary Equation 95)}$$

We take the sum over the indices k_1 and k_2 to obtain:

$$\delta\lambda_0^2 = \frac{\sum_{k,l} C^{-1}_{kl}}{\left(\sum_{k,l} C^{-1}_{kl} \right)^2}, \qquad \text{(Supplementary Equation 96)}$$

which simplifies to equation (30) in the main text.

Supplementary Note 6 Sensory multiplexing for the two-dimensional elastic sheet

In this Supplementary Note, we determine the covariance matrices for the sensory multiplexing protocols described in the main text. For the collection of boundary probes, inserting equation (9) into the definition of the covariance matrix yields:

$$C_{kl} = \Delta_\lambda \xi^D \frac{\int \psi_k(\boldsymbol{r}) \psi_l(\boldsymbol{r}) d\boldsymbol{r}}{\left(\int \psi_k(\boldsymbol{r}) d\boldsymbol{r} \int \psi_l(\boldsymbol{r}) d\boldsymbol{r} \right)}. \qquad \text{(Supplementary Equation 97)}$$

The probe fields in equations (31) and (32) give rise to the following probe potentials:

$$V^{(+)}_{f,k}(\boldsymbol{r}) = V^{(+)}_{w,k}(\boldsymbol{r}) = \frac{1}{k} \left(\frac{r}{a} \right)^k \cos(k\theta). \qquad \text{(Supplementary Equation 98)}$$

$$V_{f,k}^{(-)}(\boldsymbol{r}) = V_{w,k}^{(-)}(\boldsymbol{r}) = \frac{1}{k}\left(\frac{a}{r}\right)^k \cos(k\theta). \quad \text{(Supplementary Equation 99)}$$

in the interior and the exterior, respectively, for $k \geq 1$, and where $\nabla^2 V_{f,k}(\boldsymbol{r}) = f_k(\boldsymbol{r})$ and $\nabla^2 V_{w,k}(\boldsymbol{r}) = w_k(\boldsymbol{r})$ (see Supplementary Note 1). These probe potentials cast probe intensities $\psi_k(\boldsymbol{r})$ that are proportional to the diagonal terms in the matrix $\mathcal{M}_{kl}^{(\pm)}$ that appears in Supplementary Note 4. Indeed, for each value of $k_{\max}$, the following identification:

$$p_k \to kP_{kk}, \quad \text{(Supplementary Equation 100)}$$

maps $\Psi(\boldsymbol{r})$ for the boundary probes in Sensory multiplexing can significantly improve the precision of sensing of the main text onto a $\psi(\boldsymbol{r})$ for the convex relaxation in Supplementary Note 4. A similar mapping can be done for sensory multiplexing protocols that incorporate contributions from pairs of modes with unequal mode numbers, which we did not consider in the main text. Such contributions correspond to the off-diagonal elements of $\mathcal{M}_{kl}^{(\pm)}$. Thus, for a boundary probe, the possible $\Psi(\boldsymbol{r})$ that can be achieved by sensory multiplexing are equivalent to the possible $\psi(\boldsymbol{r})$ for the convex relaxation in Supplementary Note 4.

Based on this mapping, the results of Supplementary Note 4 imply that the above sensory multiplexing protocol achieves a fractional uncertainty that saturates to a constant value $\delta\lambda_0/\lambda_0 \approx \eta/\sqrt{\pi}$ in the asymptotic limit $k_{\max} \gg 1$. This saturation occurs because different boundary probes are correlated via their overlapping probe intensities $\psi_k(\boldsymbol{r})$ in the interior, which partially censors the information that the sensor can extract from the exterior. Thus, the sensor must account for these correlations in order to access the full extent of the information available from the exterior. To that end, the sensor can adjust each estimator to nullify their effective probe intensities in the interior. Specifically, the sensor can perform sensory multiplexing using the adjusted estimators $\hat{\zeta}_{0,k}$ given by the weighted sum of each estimator $\hat{\lambda}_{0,k}$ and an appropriately chosen companion estimator $\hat{\hat{\lambda}}_{0,k}$:

$$\hat{\zeta}_{0,k} = q_k \hat{\lambda}_{0,k} + \tilde{q}_k \hat{\hat{\lambda}}_{0,k}. \quad \text{(Supplementary Equation 101)}$$

Imposing the additional constraint $q_k + \tilde{q}_k = 1$ ensures that the adjusted estimator $\hat{\zeta}_{0,k}$ is unbiased. Here, the companion estimator $\hat{\hat{\lambda}}_{0,k}$ is given by:

$$\hat{\hat{\lambda}}_{0,k} = \frac{\int \tilde{\psi}_k(\boldsymbol{r})\lambda(\boldsymbol{r})d\boldsymbol{r}}{\int \tilde{\psi}_k(\boldsymbol{r})d\boldsymbol{r}}, \quad \text{(Supplementary Equation 102)}$$

where $\tilde{\psi}_k(\boldsymbol{r}) \equiv \nabla\tilde{V}_{f,k}(\boldsymbol{r}) \cdot \nabla\tilde{V}_{w,k}(\boldsymbol{r})$ is the probe intensity for the companion probe k. To cancel out the interior, we take $\tilde{V}_{f,k}(\boldsymbol{r})$ and $\tilde{V}_{w,k}(\boldsymbol{r})$ to be given by:

$$\tilde{V}_{f,k}(\boldsymbol{r}) \sim r - a, \quad \text{(Supplementary Equation 103)}$$

$$\tilde{V}_{w,k}(\boldsymbol{r}) \sim \left(\frac{r}{a}\right)^{2k-1} - 1, \quad \text{(Supplementary Equation 104)}$$

for $r < a$ and 0 otherwise. These probe potentials can be generated by the probe fields $\tilde{f}_k(\boldsymbol{r}) = \nabla^2\tilde{V}_{f,k}(\boldsymbol{r})$ and $\tilde{w}_k(\boldsymbol{r}) = \nabla^2\tilde{V}_{w,k}(\boldsymbol{r})$, and they combine to yield the following probe intensities $\tilde{\psi}_k(\boldsymbol{r})$:

$$\tilde{\psi}_k(\boldsymbol{r}) = \begin{cases} 2k\left(\frac{r}{a}\right)^{2k-2}, & r < a. \\ 0, & r > a. \end{cases} \quad \text{(Supplementary Equation 105)}$$

Inserting the probe intensities in equation (33) and Supplementary Equation 105 into the estimators in Supplementary Equation 101 results in:

$$\hat{\zeta}_{0,k} = \frac{\int \Psi_k(\boldsymbol{r})\lambda(\boldsymbol{r})d\boldsymbol{r}}{\int \Psi_k(\boldsymbol{r})d\boldsymbol{r}}, \quad \text{(Supplementary Equation 106)}$$

where the effective probe intensities $\Psi_k(\boldsymbol{r})$ are given by:

$$\Psi_k(\boldsymbol{r}) \sim \begin{cases} q_k k \left(\frac{r}{a}\right)^{2k-2} + \tilde{q}_k 2k \left(\frac{r}{a}\right)^{2k-2}, & r < a. \\ q_k k \left(\frac{r}{a}\right)^{-2k-2}, & r > a. \end{cases} \quad \text{(Supplementary Equation 107)}$$

Thus, to nullify the interiors of $\Psi_k(\boldsymbol{r})$, we must have $q_k + 2\tilde{q}_k = 0$. Combining this equation with the constraint for unbiasedness leads to $q_k = 2$ and $\tilde{q}_k = -1$ for all values of k. Inserting these values into the above equation results in the paired probes referred to in Sensory multiplexing can significantly improve the precision of sensing:

$$\Psi_k(\boldsymbol{r}) \sim \begin{cases} 0, & r < a. \\ 2k \left(\frac{r}{a}\right)^{-2k-2}, & r > a. \end{cases} \quad \text{(Supplementary Equation 108)}$$

Finally, the sensor may extract additional information by uniformly sampling the material constant field in its interior. To do so, the sensor may adjust the coefficient $\tilde{q}_1$. In this case, the optimal all-inclusive effective probe intensity $\Psi(\boldsymbol{r})$ is given by:

$$\Psi(\boldsymbol{r}) = \sum_{k=1}^{k_{\max}} p_k \frac{\psi_k(\boldsymbol{r})}{\int \psi_k(\boldsymbol{r})d\boldsymbol{r}} + \sum_{k=1}^{k_{\max}} \tilde{p}_k \frac{\tilde{\psi}_k(\boldsymbol{r})}{\int \tilde{\psi}_k(\boldsymbol{r})d\boldsymbol{r}}, \quad \text{(Supplementary Equation 109)}$$

for appropriate values of p_k and $\tilde{p}_k$. Alternatively, the sensor may incorporate an unpaired probe $\tilde{\psi}_1(\boldsymbol{r})$ given by equation (35) for $k = 1$ into the sensory multiplexing protocol. This unpaired probe corresponds to the following optimal estimator $\hat{\zeta}_{0,0}$:

$$\hat{\zeta}_{0,0} = \frac{\int \tilde{\psi}_1(\boldsymbol{r})\lambda(\boldsymbol{r})d\boldsymbol{r}}{\int \tilde{\psi}_1(\boldsymbol{r})d\boldsymbol{r}}. \quad \text{(Supplementary Equation 110)}$$

Combining this unpaired probe with the paired probes described by Supplementary Equation 108 results in the following all-inclusive effective probe intensity $\Psi(\boldsymbol{r})$:

$$\Psi(\boldsymbol{r}) = p_0 \frac{\tilde{\psi}_1(\boldsymbol{r})}{\int \tilde{\psi}_1(\boldsymbol{r})d\boldsymbol{r}} + \sum_{k=1}^{k_{\max}} p_k \frac{\Psi_k(\boldsymbol{r})}{\int \Psi_k(\boldsymbol{r})d\boldsymbol{r}}, \quad \text{(Supplementary Equation 111)}$$

where $p_k = \sum_l C_{kl}^{-1}$ with $C_{kl} \equiv \langle(\hat{\zeta}_{0,k} - \lambda_0)(\hat{\zeta}_{0,l} - \lambda_0)\rangle$. The all-inclusive effective probe intensities given by Supplementary Equation 109 and Supplementary Equation 111 are equivalent in that their configurations are identical and that they result in the same fractional uncertainty. However, their sensory multiplexing protocols are physically distinct in that the latter contains one additional probe. In Supplementary Note 7, we demonstrate that this $\Psi(\boldsymbol{r})$ exhaustively probes the full extent of the information available to the sensor.

Supplementary Note 7 A fundamental, physical limit to $\delta\lambda_0/\lambda_0$ for the elastic sheet

In this Supplementary Note, we prove that the adjusted sensory multiplexing protocol presented in Sensory multiplexing can significantly improve the precision of sensing achieves the smallest possible $\delta\lambda_0/\lambda_0$ among all protocols that employ probe fields confined to a region $r \leq a$. To do so, we consider a sensory multiplexing protocol containing an arbitrary number of arbitrarily complicated probes. To determine the smallest $\delta\lambda_0/\lambda_0$ for such a protocol, we start by imagining a convex relaxation of $\Psi(\boldsymbol{r})$ that generalizes the convex relaxation in Supplementary Note 4. Specifically, we expand the space of possible $\Psi(\boldsymbol{r})$ to allow an arbitrary configuration in the interior combined

with any configuration in the exterior that can be generated by the convex relaxation of equation (23). This convex relaxation of $\Psi(\boldsymbol{r})$ expands its possible configurations to include some that cannot be cast by a single volume probe. It follows that the minimum fractional uncertainty $\delta\lambda_{0,\text{low}}/\lambda_0$ for this convex relaxation provides a theoretical lower bound on $\delta\lambda_0/\lambda_0$. Moreover, provided that a sequence of multiple physical measurements can realize this lower bound, $\delta\lambda_{0,\text{low}}/\lambda_0$ provides a physical limit to $\delta\lambda_0/\lambda_0$.

We determine $\delta\lambda_{0,\text{low}}/\lambda_0$ by first separating the action S for the variance $\delta\lambda_0^2$ into the sum $S = S_{\text{int}} + S_{\text{ext}}$ of contributions from the interior $\mathcal{R}_{\text{int}}$ and the exterior $\mathcal{R}_{\text{ext}}$. The contribution from the interior is given by:

$$S_{\text{int}} = \int_{\mathcal{R}_{\text{int}}} \left(\frac{1}{2}\Psi(\boldsymbol{r})^2 - \gamma\Psi(\boldsymbol{r}) \right) d\boldsymbol{r}, \qquad \text{(Supplementary Equation 112)}$$

where the integral is taken over the interior $\mathcal{R}_{\text{int}}$ of the sensor ($r < a$) and γ is a Lagrange multiplier that fixes $\int \Psi(\boldsymbol{r})d\boldsymbol{r}$. This action is minimized by $\Psi(\boldsymbol{r}) = \gamma$. For the exterior, the action is (in units of $a = 1$):

$$S_{\text{ext}} = \frac{1}{2}\int_{\mathcal{R}_{\text{ext}}} \left(\sum_{k,l=1}^{k_{\max}} P_{kl}\mathcal{M}_{kl}^{(1,-)} + Q_{kl}\mathcal{M}_{kl}^{(2,-)} \right)^2 d\boldsymbol{r} - \gamma \sum_{k=1}^{k_{\max}} \pi k P_{kk}, \qquad \text{(Supplementary Equation 113)}$$

where γ is the same Lagrange multiplier as in Supplementary Equation 112, the matrices $\mathcal{M}_{kl}^{(1,-)}$ and $\mathcal{M}_{kl}^{(2,-)}$ are the same as in Supplementary Note 4, and P_{kl} and Q_{kl} are arbitrary real matrices. To determine the optimal $\Psi(\boldsymbol{r})$ in the exterior, we must minimize S_{ext} over P_{kl} and Q_{kl}. To that end, our treatment of the convex relaxation in Supplementary Note 4 implies that the optimal $\Psi(\boldsymbol{r})$ in the exterior must only receive contributions from the diagonal terms of $\mathcal{M}_{kl}^{(1,-)}$. These contributions can be mapped onto the paired probes $\Psi_k(\boldsymbol{r})$ in Supplementary Equation 111 via $kP_{kk} \to p_k$. Finally, we observe that the all-inclusive $\Psi(\boldsymbol{r})$ described in Sensory multiplexing can significantly improve the precision of sensing simultaneously determines the optimal p_k and γ (which corresponds to p_0 in Supplementary Equation 111). Therefore, this $\Psi(\boldsymbol{r})$ minimizes the convex relaxation of S, and thereby attains the smallest physically possible $\delta\lambda_0/\lambda_0$. This proof generalizes to $D = 3$ in a straightforward manner.

Supplementary Note 8 Sensory multiplexing for a three-dimensional elastic medium

In this Supplementary Note, we quantify the precision of a sensor that can perform multiple probes of a three-dimensional, elastic medium. For simplicity, we constrain the medium to deform as a scalar $u(\boldsymbol{r})$ at each point in space, analogous to our treatment of the two-dimensional elastic sheet. Physically, this medium corresponds to an anisotropic elastic solid constrained to deform along a single direction. The internal energy of such an elastic solid is given by:

$$E = \frac{1}{2}\int \lambda(\boldsymbol{r})\nabla u(\boldsymbol{r}) \cdot \nabla u(\boldsymbol{r})d\boldsymbol{r}. \qquad \text{(Supplementary Equation 114)}$$

Here, as for the Winkler foundation and the elastic sheet, we take $\lambda(\boldsymbol{r})$ to be a Gaussian random field with mean λ_0, variance $\Delta_\lambda \ll \lambda_0^2$, and spatial correlations over a scale ξ. As before, we take the sensor to interact with the medium within a radius a by first applying a stimulus field $f(\boldsymbol{r})$ as in equation (4), and then measuring an integrated response m as in equation (5).

In what follows, we will determine the fractional uncertainty for a sensory multiplexing protocol applied to this elastic medium in the asymptotic limit of fine spatial resolution. Motivated by the results in the main text for the case of the elastic sheet, we take each probe i to apply the following probe fields:

$$f_i(\boldsymbol{r}) \sim \delta(r-a)Y_{\ell_i m_i}(\theta,\varphi), \qquad \text{(Supplementary Equation 115)}$$

$$w_i(\boldsymbol{r}) \sim \delta(r-a)Y^*_{\ell_i m_i}(\theta,\varphi), \qquad \text{(Supplementary Equation 116)}$$

where Y_{ℓ_i,m_i} are spherical harmonics of degree ℓ_i and order m_i and $Y^*_{\ell_i,m_i}$ are their complex conjugates. To be concrete, we choose the prefactors of the probe fields such that the probe potentials are given by:

$$V_{f,i}^{(+)}(\boldsymbol{r}) = \frac{r^{\ell} Y_{\ell_i m_i}(\theta,\varphi)}{(2\ell+1)a^{\ell-1}}. \qquad \text{(Supplementary Equation 117)}$$

$$V_{w,i}^{(+)}(\boldsymbol{r}) = \frac{r^{\ell} Y^*_{\ell_i m_i}(\theta,\varphi)}{(2\ell+1)a^{\ell-1}}. \qquad \text{(Supplementary Equation 118)}$$

$$V_{f,i}^{(-)}(\boldsymbol{r}) = \frac{a^{\ell+2} Y_{\ell_i m_i}(\theta,\varphi)}{(2\ell+1)r^{\ell+1}}. \qquad \text{(Supplementary Equation 119)}$$

$$V_{w,i}^{(-)}(\boldsymbol{r}) = \frac{a^{\ell+2} Y^*_{\ell_i m_i}(\theta,\varphi)}{(2\ell+1)r^{\ell+1}}, \qquad \text{(Supplementary Equation 120)}$$

in the interior and exterior, respectively (see Supplementary Note 1 for definition of probe potentials). Moreover, we assume that the sensor executes such probes for all possible values of m and ℓ up to a maximum degree $\ell_{\max}$. For this sensory geometry, we define the sensor resolution d to be inversely proportional to $\ell_{\max}$:

$$d \sim \frac{a}{\ell_{\max}}. \qquad \text{(Supplementary Equation 121)}$$

As before for the two-dimensional elastic sheet, the MVUE of λ_0 for the above sensory multiplexing protocol is given by the best linear unbiased sum of the estimators $\hat{\lambda}_{0,i}$ of individual probes:

$$\hat{\lambda}_0 = \sum_i p_i \hat{\lambda}_{0,i}, \qquad \text{(Supplementary Equation 122)}$$

where the estimator weights p_i are the following normalized sums over the rows of the inverse C^{-1}_{ij} of the covariance matrix:

$$p_i = \frac{\sum_j C^{-1}_{ij}}{\sum_{ij} C^{-1}_{ij}}. \qquad \text{(Supplementary Equation 123)}$$

The covariance matrix C_{ij} is defined by Supplementary Equation 97. The variance $\delta\lambda_0^2$ of the estimator $\hat{\lambda}_0$ is given by:

$$\delta\lambda_0^2 = \sum_{ij} p_i p_j C_{ij}. \qquad \text{(Supplementary Equation 124)}$$

To calculate the covariance matrix, we insert the probe potentials into Supplementary Equation 97, which results in:

$$C_{ij} = \Delta_\lambda \xi^3 \left(\int_{\mathcal{R}_{\text{int}}} \frac{\psi_i^{(+)}(\boldsymbol{r})\psi_j^{(+)}(\boldsymbol{r})}{s_i s_j} d\boldsymbol{r} + \int_{\mathcal{R}_{\text{ext}}} \frac{\psi_i^{(-)}(\boldsymbol{r})\psi_j^{(-)}(\boldsymbol{r})}{s_i s_j} d\boldsymbol{r} \right), \qquad \text{(Supplementary Equation 125)}$$

where

$$\psi_i^{(\pm)}(\boldsymbol{r}) = \nabla V_{w,i}^{(\pm)}(\boldsymbol{r}) \cdot \nabla V_{f,i}^{\pm()}(\boldsymbol{r}), \qquad \text{(Supplementary Equation 126)}$$

and

$$s_i = \int_{\mathcal{R}_{\text{int}}} \psi_i^{(+)}(\boldsymbol{r}) d\boldsymbol{r} + \int_{\mathcal{R}_{\text{ext}}} \psi_i^{(-)}(\boldsymbol{r}) d\boldsymbol{r}, \qquad \text{(Supplementary Equation 127)}$$

are the normalizing constants given by:

$$s_i = \frac{a^3}{2\ell + 1}. \quad \text{(Supplementary Equation 128)}$$

Inserting the covariance matrix given by Supplementary Equation 125 into Supplementary Equation 124 results in:

$$\delta\lambda_0^2 = \Delta_\lambda \xi^3 \sum_{ij} p_i p_j \left(\int_{\mathcal{R}_{(+)}} \frac{\psi_i^{(+)}(\boldsymbol{r})\psi_j^{(+)}(\boldsymbol{r})}{s_i s_j} d\boldsymbol{r} + \int_{\mathcal{R}_{\text{ext}}} \frac{\psi_i^{(-)}(\boldsymbol{r})\psi_j^{(-)}(\boldsymbol{r})}{s_i s_j} d\boldsymbol{r} \right), \quad \text{(Supplementary Equation 129)}$$

which can be separated into the sum $\delta\lambda_0^2 = \delta\lambda_{0,\text{int}}^2 + \delta\lambda_{0,\text{ext}}^2$ of contributions from the interior and the exterior:

$$\delta\lambda_{0,\text{int}}^2 = \Delta_\lambda \xi^3 \sum_{ij} p_i p_j \int_{\mathcal{R}_{\text{int}}} \frac{\psi_i^{(+)}(\boldsymbol{r})\psi_j^{(+)}(\boldsymbol{r})}{s_i s_j} d\boldsymbol{r}, \quad \text{(Supplementary Equation 130)}$$

$$\delta\lambda_{0,\text{ext}}^2 = \Delta_\lambda \xi^3 \sum_{ij} p_i p_j \int_{\mathcal{R}_{\text{ext}}} \frac{\psi_i^{(-)}(\boldsymbol{r})\psi_j^{(-)}(\boldsymbol{r})}{s_i s_j} d\boldsymbol{r}. \quad \text{(Supplementary Equation 131)}$$

To evaluate these integrals, we first consider the contribution $\delta\lambda_{0,\text{ext}}^2$ from the exterior. The above equation can be expressed in terms of the probe potentials as follows:

$$\delta\lambda_{0,\text{ext}}^2 = \Delta_\lambda \xi^3 \sum_{\ell_i=0}^{\ell_{\max}} \sum_{\ell_j=0}^{\ell_{\max}} \sum_{m_i=-\ell_i}^{\ell_i} \sum_{m_j=-\ell_j}^{\ell_j} p_i p_j \int_{\mathcal{R}_{\text{ext}}} \frac{a^{2\ell_i+1}}{(2\ell_i+1)} \nabla\left(\frac{Y_{\ell_i m_i}}{r^{\ell_i+1}}\right) \cdot \nabla\left(\frac{Y_{\ell_i m_i}^*}{r^{\ell_i+1}}\right) \frac{a^{2\ell_j+1}}{(2\ell_j+1)} \nabla\left(\frac{Y_{\ell_j m_j}}{r^{\ell_j+1}}\right) \cdot \nabla\left(\frac{Y_{\ell_j m_j}^*}{r^{\ell_j+1}}\right) d\boldsymbol{r}.$$

(Supplementary Equation 132)

We swap the order of the sums and the integration to obtain:

$$\delta\lambda_0^2 = \Delta_\lambda \xi^3 \int_{\mathcal{R}_{\text{ext}}} \left(\sum_{\ell_i=0}^{\ell_{\max}} \sum_{m_i=-\ell_i}^{\ell_i} \frac{p_i a^{2\ell_i+1}}{(2\ell_i+1)} \nabla\left(\frac{Y_{\ell_i m_i}}{r^{\ell_i+1}}\right) \cdot \nabla\left(\frac{Y_{\ell_i m_i}^*}{r^{\ell_i+1}}\right) \right)^2 d\boldsymbol{r}. \quad \text{(Supplementary Equation 133)}$$

To compute the sum over the spherical harmonic orders m_i, we must know the values of p_i. Numerical minimization of the variance indicates that the values of these coefficients are independent of the spherical harmonic orders m_i. Using this ansatz, we can express the values of these coefficients as:

$$p_i = \frac{p_{\ell_i}}{2\ell_i + 1}, \quad \text{(Supplementary Equation 134)}$$

where p_{ℓ_i} is a constant that depends on the degree ℓ_i of the probe i and $\sum_i p_{\ell_i} = 1$. Inserting this expression for the weights in Supplementary Equation 133 yields:

$$\delta\lambda_{0,\text{ext}}^2 = \Delta_\lambda \xi^3 \int_{\mathcal{R}_{\text{ext}}} \left(\sum_{\ell_i=0}^{\ell_{\max}} \sum_{m_i=-\ell_i}^{\ell_i} \frac{p_{\ell_i} a^{2\ell_i+1}}{(2\ell_i+1)^2} \nabla\left(\frac{Y_{\ell_i m_i}}{r^{\ell_i+1}}\right) \cdot \nabla\left(\frac{Y_{\ell_i m_i}^*}{r^{\ell_i+1}}\right) \right)^2 d\boldsymbol{r}. \quad \text{(Supplementary Equation 135)}$$

To proceed, we employ the closure relationship for the sum of the spherical harmonic orders[7], which implies the following identity:

$$\sum_m \nabla\left(f(r) Y_{\ell m}\right) \cdot \nabla\left(f(r) Y_{\ell m}^*\right) = \frac{2\ell+1}{4\pi} \left(\frac{\ell(\ell+1) f(r)^2}{r^2} + f'(r)^2 \right). \quad \text{(Supplementary Equation 136)}$$

Using this identity, we take the sum over the orders m_i in Supplementary Equation 135 to find:

$$\delta\lambda_{0,\mathrm{ext}}^2 = \Delta_\lambda \xi^3 \int_{\mathcal{R}_{\mathrm{ext}}} \left(\sum_{\ell_i} p_{\ell_i} \frac{(\ell_i+1)a^{2\ell_i+1}}{4\pi r^{2\ell_i+4}} \right)^2 d\boldsymbol{r}. \quad \text{(Supplementary Equation 137)}$$

We now expand the sum and perform the integral to obtain:

$$\delta\lambda_{0,\mathrm{ext}}^2 = \frac{\Delta_\lambda}{4\pi} \left(\frac{\xi}{a}\right)^3 \sum_{\ell_i \ell_j} p_{\ell_i} p_{\ell_j} \frac{(\ell_i+1)(\ell_j+1)}{2\ell_i+2\ell_j+5}. \quad \text{(Supplementary Equation 138)}$$

A similar calculation can be performed for the interior, which yields:

$$\delta\lambda_{0,\mathrm{int}}^2 = \frac{\Delta_\lambda}{4\pi} \left(\frac{\xi}{a}\right)^3 \sum_{\ell_i \ell_j} p_{\ell_i} p_{\ell_j} \frac{\ell_i \ell_j}{2\ell_i+2\ell_j-1}. \quad \text{(Supplementary Equation 139)}$$

Adding up the contributions $\delta\lambda_{0,\mathrm{int}}^2$ and $\delta\lambda_{0,\mathrm{ext}}^2$ from the interior and the exterior results in:

$$\delta\lambda_0^2 = \frac{\Delta_\lambda}{4\pi} \left(\frac{\xi}{a}\right)^3 \sum_{\ell_i \ell_j} p_{\ell_i} p_{\ell_j} \tilde{C}_{ij}, \quad \text{(Supplementary Equation 140)}$$

where $\tilde{C}_{ij}$ is a dimensionless matrix given by:

$$\tilde{C}_{ij} = \frac{\ell_i \ell_j}{2\ell_i+2\ell_j-1} + \frac{(\ell_i+1)(\ell_j+1)}{2\ell_i+2\ell_j+5}. \quad \text{(Supplementary Equation 141)}$$

In terms of this matrix, the variance $\delta\lambda_0^2$ is given by:

$$\delta\lambda_0^2 = \frac{\Delta_\lambda}{4\pi} \left(\frac{\xi}{a}\right)^3 \left(\sum_{ij} \tilde{C}_{ij}^{-1} \right)^{-1}. \quad \text{(Supplementary Equation 142)}$$

As before for the two-dimensional elastic sheet, we find that the contributions from the interior introduce correlations among the probes that limit the amount of information that can be extracted. To remove these unnecessary correlations, we consider the companion probes given by:

$$V_{f,\ell}(\boldsymbol{r}) \sim a - r, \quad \text{(Supplementary Equation 143)}$$

$$V_{w,\ell}(\boldsymbol{r}) \sim \left(\frac{r}{a}\right)^{2\ell-2} - 1. \quad \text{(Supplementary Equation 144)}$$

We combine these companion probes with those of the original protocol, as in Supplementary Note 6, to obtain the following adjusted matrix $\tilde{\tilde{C}}_{ij}$:

$$\tilde{\tilde{C}}_{ij} = \frac{(2\ell_i+1)(2\ell_j+1)}{2\ell_i+2\ell_j+5}, \quad \text{(Supplementary Equation 145)}$$

which has a variance given by:

$$\delta\lambda_0^2 = \frac{\Delta_\lambda}{4\pi} \left(\frac{\xi}{a}\right)^3 \left(\sum_{ij} \tilde{\tilde{C}}_{ij}^{-1} \right)^{-1}. \quad \text{(Supplementary Equation 146)}$$

Inserting the adjusted matrix $\tilde{\tilde{C}}_{ij}$ in Supplementary Equation 145 into the above equation results in:

$$\delta\lambda_0^2 = \frac{\Delta_\lambda}{4\pi}\left(\frac{\xi}{a}\right)^3\left(\sum_{k=1}^{\ell_{\max}+1}\Upsilon(k)\right)^{-1}, \qquad \text{(Supplementary Equation 147)}$$

where $\Upsilon(k)$ is given by:

$$\Upsilon(k) = (4k+5)\frac{(k+1)!\,(k+1)!}{(k+1/2)!\,(k+1/2)!}. \qquad \text{(Supplementary Equation 148)}$$

In the limit $k \to \infty$, this function scales as $\Upsilon(k) \sim k^2$ (by Stirling's approximation). Thus, for a large maximum degree $\ell_{\max} \to \infty$, the sum in Supplementary Equation 147 approaches:

$$\delta\lambda_0^2 \sim \frac{\Delta_\lambda}{4\pi}\left(\frac{\xi}{a}\right)^3\left(\sum_{k=\ell_0}^{\ell_{\max}+1}k^2\right)^{-1}, \qquad \text{(Supplementary Equation 149)}$$

where $\ell_0 \gg 1$. We compute the sum of these consecutive squares to find:

$$\delta\lambda_0^2 \sim \frac{\Delta_\lambda}{4\pi}\left(\frac{\xi}{a}\right)^3\left(\frac{(2\ell_{\max}+1)\ell_{\max}(\ell_{\max}+1)}{6} - \frac{(2\ell_0+1)\ell_0(\ell_0+1)}{6}\right)^{-1}. \qquad \text{(Supplementary Equation 150)}$$

Finally, we take the limit $\ell_{\max} \gg \ell_0$ to obtain the following scaling for the fractional uncertainty:

$$\frac{\delta\lambda_0}{\lambda_0} \sim \left(\frac{\Delta_\lambda}{\lambda_0^2}\right)^{1/2}\left(\frac{d}{a}\right)^{3/2}\left(\frac{\xi}{a}\right)^{3/2}, \qquad \text{(Supplementary Equation 151)}$$

which matches equation (39) in the main text for $D = 3$.

Supplementary Note 9 Sensory multiplexing is robust to the omission of modes

In the main text, we considered sensory multiplexing protocols that harnessed all possible mode pairs up to a maximum mode number $k_{\max}$. How does the precision of the sensor change if this assumption is violated? To gain insight into this question, we consider a sensor that executes the paired probes in Sensory multiplexing can significantly improve the precision of sensing starting from an initial mode number $k_{\min}$ up to a maximum mode number $k_{\max}$. For this sensory multiplexing protocol, the covariance matrix is again given by equation (37). Inserting the inverse of this matrix into equation (30) and taking the sums to range over the included probes results in the variance:

$$\delta\lambda_0^2 = 2\Delta_\lambda\left(\frac{\xi}{a}\right)^2\frac{k_{\min}^2}{(k_{\max}+k_{\min}+1)(k_{\max}-k_{\min}+1)}. \qquad \text{(Supplementary Equation 152)}$$

This variance increases with $k_{\min}$, which indicates that the precision of the sensor worsens as mode pairs are omitted. Nevertheless, in the limit $k_{\max} \gg k_{\min}$, the scaling of the fractional uncertainty with the sensor's resolution is again given by equation (39). Moreover, we have explored variants of the above protocol that consist of omitting intermediate mode pairs, and found that they also obey the scaling in equation (39) for $k_{\max} \gg k_{\min}$. Taken together, our results suggest that the details of the measurement protocol do not affect the scaling of $\delta\lambda_0/\lambda_0$ with d, provided that the sensor probes a sufficiently large number of mode pairs.

Supplementary Note 10 A numerical lower bound on $\delta\lambda_0/\lambda_0$ for volume probes

To what extent do interference effects limit a sensor that can apply a single, arbitrary probe within its volume? To gain insight into this question, we extended the numerical approach discussed in Probe field interference limits the channel capacity of sensing to account for such volume probes.

Probe fields containing bulk modes provide $\sim k_{\max}^{16}$ contributions to $\delta\lambda_0^2$. This rapid scaling drastically limits the scope of conventional numerical minimization. To maximize the reach of our computational capabilities, we considered a constraint relaxation of $\delta\lambda_0/\lambda_0$ that allows us to treat the interior and exterior of the sensor separately. Specifically, we separate the action S for the variance $\delta\lambda_0^2$ into the sum $S = S_{\text{int}} + S_{\text{ext}}$, where

$$S_{\text{int}} = \int_{\mathcal{R}_{\text{int}}} \left(\frac{1}{2}\psi(\boldsymbol{r})^2 - \gamma\psi(\boldsymbol{r}) \right) d\boldsymbol{r}, \qquad \text{(Supplementary Equation 153)}$$

and

$$S_{\text{ext}} = \Delta_\lambda \xi^D \sum_{k,l,m,n} B_{kl} B_{mn} \mathbb{T}_{klmn}, \qquad \text{(Supplementary Equation 154)}$$

where $B_{kl} \sim B_k^{(f)} B_l^{(w)}$ is the outer product of two complex vectors $B_k^{(f)}$ and $B_k^{(w)}$ that satisfy $B_{-k}^{(f)} = B_k^{(f)*}$ and $B_{-k}^{(w)} = B_k^{(w)*}$, and $\mathbb{T}_{klmn}$ is a highly structured, fourth-order tensor:

$$\mathbb{T}_{klmn} = 2\pi a^2 \frac{\delta_{k-l+m-n,0}\, y_{klmn}}{(x_{klmn} + 2)}. \qquad \text{(Supplementary Equation 155)}$$

Here, $\delta_{i,j}$ is the Kronecker delta function, $x_{klmn} = |k| + |l| + |m| + |n|$, and $y_{klmn} = (kl + |kl|)(mn + |mn|)$. We then minimize S_{int} and S_{ext} individually, disregarding the constraint that the probe potentials must be continuous across the sensor's boundary $\mathcal{B}$, but still imposing the constraint $\int \psi(\boldsymbol{r}) d\boldsymbol{r} = 1$. It follows that the sum of the minima of S_{int} and S_{ext} provide a lower bound $\delta\lambda_{0,\text{low}}^2$ on the minimum of S (see subsection below).

The contribution S_{int} is minimized by a uniform probe intensity $\psi(\boldsymbol{r}) = \gamma$ in $\mathcal{R}_{\text{int}}$, as we found in Supplementary Note 7. For the exterior, the probe potentials are completely determined by their values on the boundary, which enabled us to employ the same numerical optimization scheme as for the boundary probes (see Methods). For all values of $k_{\max} > 1$, we found that the resulting probe potentials were dominated by the dipole-dipole and quadrupole-quadrupole pairs, with higher order modes observed for $k_{\max} > 8$ (see Fig. 3 of the main text). Moreover, in this case, the theoretical lower bound $\delta\lambda_{0,\text{low}}/\lambda_0$ does not provide a close match to the values of $\delta\lambda_0/\lambda_0$ obtained for sensory multiplexing. This discrepancy suggests that even if a sensor is capable of applying an arbitrary pair of probe fields within its volume, interferences between modes significantly restrict the information that the sensor can glean in comparison to sensory multiplexing.

A lower bound on the variance $\delta\lambda_0^2$

Here, we prove that separately minimizing $\psi(\boldsymbol{r})$ in the interior and the exterior yields a lower bound $\delta\lambda_{0,\text{low}}^2$ on the true minimum $\delta\lambda_{0,\min}^2$ of the variance $\delta\lambda_0^2$. The minimum variance $\delta\lambda_{0,\min}^2$ is given by:

$$\begin{aligned} \delta\lambda_{0,\min}^2 = \underset{V_f, V_w}{\text{minimize}} \quad & S(V_f, V_w) \\ \text{subject to} \quad & \mathcal{C}_1(V_f, V_w, V_f, V_w),\ \mathcal{C}_2(V_f, V_w), \end{aligned} \qquad \text{(Supplementary Equation 156)}$$

where $S = \int_{\mathcal{R}} (\nabla V_f \cdot \nabla V_w)^2 d\boldsymbol{r}$ is the unconstrained action for $\delta\lambda_0^2$ integrated over all of space $\mathcal{R}$, the constraint $\mathcal{C}_1(V_f^{(i)}, V_w^{(i)}, V_f^{(j)}, V_w^{(j)})$ is a function of two configurations i and j of the probe potentials that fixes the normalization of the probe intensity:

$$\int_{\mathcal{R}_{\text{int}}} (\nabla V_f^{(i)} \cdot \nabla V_w^{(i)}) d\boldsymbol{r} + \int_{\mathcal{R}_{\text{ext}}} (\nabla V_f^{(j)} \cdot \nabla V_w^{(j)}) d\boldsymbol{r} = 1, \qquad \text{(Supplementary Equation 157)}$$

and the constraint $\mathcal{C}_2(V_f, V_w)$ enforces the constraints imposed by the finite size of the probe, i.e. that:

$$\nabla^2 V_f = 0, \qquad \text{(Supplementary Equation 158)}$$

$$\nabla^2 V_w = 0, \qquad \text{(Supplementary Equation 159)}$$

for $\boldsymbol{r} \in \mathcal{R}_{\text{ext}}$. This minimization procedure yields the true, optimal probe potentials $V_f^{(A)}$ and $V_w^{(A)}$. Thus, $\delta\lambda_{0,\text{min}}^2$ is given by:

$$\delta\lambda_{0,\text{min}}^2 = S(V_f^{(A)}, V_w^{(A)}). \qquad \text{(Supplementary Equation 160)}$$

To determine a lower bound on this quantity, we start by separating the variance into the following sum:

$$S = S_{\text{int}} + S_{\text{ext}}, \qquad \text{(Supplementary Equation 161)}$$

where the contributions S_{int} and S_{ext} are given by:

$$S_{\text{int}} = \int_{\mathcal{R}_{\text{int}}} (\nabla V_f \cdot \nabla V_w)^2 d\boldsymbol{r}, \qquad \text{(Supplementary Equation 162)}$$

and

$$S_{\text{ext}} = \int_{\mathcal{R}_{\text{ext}}} (\nabla V_f \cdot \nabla V_w)^2 d\boldsymbol{r}. \qquad \text{(Supplementary Equation 163)}$$

The action S can be separated in this manner because we have assumed that the probe potentials V_f and V_w are continuous. This continuity ensures that the probe intensity $\psi(\boldsymbol{r})$ cannot diverge anywhere in space, and thereby precludes any additional contributions to the right hand side of Supplementary Equation 161 from the boundary $\mathcal{B}$. In what follows, we will show that a lower bound $\delta\lambda_{0,\text{low}}^2$ on the variance is obtained by separately minimizing the probe potentials in the interior and exterior as follows:

$$\begin{aligned} \delta\lambda_{0,\text{low}}^2 = \underset{V_f^{(i)}, V_w^{(i)}, V_f^{(j)}, V_w^{(j)}}{\text{minimize}} \quad & S_{\text{int}}(V_f^{(i)}, V_w^{(i)}) + S_{\text{ext}}(V_f^{(j)}, V_w^{(j)}) \\ \text{subject to} \quad & \mathcal{C}_1(V_f^{(i)}, V_w^{(i)}, V_f^{(j)}, V_w^{(j)}),\ \mathcal{C}_2(V_f^{(j)}, V_w^{(j)}). \end{aligned} \qquad \text{(Supplementary Equation 164)}$$

This minimization procedure yields probe potentials in the interior ($V_f^{(B)}$ and $V_w^{(B)}$) and in the exterior ($V_f^{(C)}$ and $V_w^{(C)}$). Thus, $\delta\lambda_{0,\text{low}}^2$ is given by:

$$\delta\lambda_{0,\text{low}}^2 = S_{\text{int}}(V_f^{(B)}, V_w^{(B)}) + S_{\text{ext}}(V_f^{(C)}, V_w^{(C)}). \qquad \text{(Supplementary Equation 165)}$$

Clearly, the following inequality must hold:

$$S_{\text{int}}(V_f^{(B)}, V_w^{(B)}) + S_{\text{ext}}(V_f^{(C)}, V_w^{(C)}) \leq S_{\text{int}}(V_f^{(A)}, V_w^{(A)}) + S_{\text{ext}}(V_f^{(A)}, V_w^{(A)}), \qquad \text{(Supplementary Equation 166)}$$

because taking the probe potentials in Supplementary Equation 164 to be $V_f^{(i)} \to V_f^{(A)}$, $V_w^{(i)} \to V_w^{(A)}$, $V_f^{(j)} \to V_f^{(A)}$, and $V_w^{(j)} \to V_w^{(A)}$ satisfies the constraints and thereby provides a candidate solution for $\delta\lambda_{0,\text{low}}^2$. Accordingly, probe potentials that do not satisfy Supplementary Equation 166 can only increase $\delta\lambda_{0,\text{low}}^2$, and so would not satisfy Supplementary Equation 164. Thus, the above inequality, taken together with Supplementary Equation 160, implies:

$$\delta\lambda_{0,\text{low}}^2 \leq \delta\lambda_{0,\text{min}}^2. \qquad \text{(Supplementary Equation 167)}$$

Supplementary Note 11 Modeling biomechanical sensing

In this Supplementary Note, we generalize our theoretical framework to an elastic medium, and we calculate the fractional uncertainty $\delta\mu_0/\mu_0$ of a model cellular probe of stiffness. The internal energy of the elastic medium described in The precision of biomechanical sensing is given by:

$$E = \int \mu(\boldsymbol{r}) \left(\frac{1}{2}\partial_i u_k(\boldsymbol{r})\partial_i u_k(\boldsymbol{r}) + \frac{1}{2}\partial_i u_k(\boldsymbol{r})\partial_k u_i(\boldsymbol{r}) + \frac{\varsigma}{2}\partial_i u_i(\boldsymbol{r})\partial_k u_k(\boldsymbol{r}) \right) d\boldsymbol{r}, \quad \text{(Supplementary Equation 168)}$$

where $u_i(\boldsymbol{r})$ is the deformation vector field, $\varsigma = 2\sigma/(1-2\sigma)$ is a constant, and repeated indices imply summation from 1 to 3 over the indexed terms. We model the cell as an idealized stiffness-measuring device that first applies a force vector field $f_i(\boldsymbol{r})$:

$$\delta E = -\int f_i(\boldsymbol{r}) u_i(\boldsymbol{r}) d\boldsymbol{r}. \quad \text{(Supplementary Equation 169)}$$

Once the medium reaches mechanical equilibrium, we assume that the cell transduces the following integrated response m:

$$m = \int w_i(\boldsymbol{r}) u_i(\boldsymbol{r}) d\boldsymbol{r}, \quad \text{(Supplementary Equation 170)}$$

where $w_i(\boldsymbol{r})$ is a weight vector field. In what follows, we will determine the optimal estimator $\hat{\mu}_0$ for this medium, and we will estimate the precision with which a cell can infer μ_0 based on m and prior knowledge of all other model parameters (including σ).

(i) The probe intensity for biomechanical sensing.

Taking the variation of the internal energy given by the sum of Supplementary Equation 168 and Supplementary Equation 169 with respect to u_i yields:

$$\delta_{i,k}\partial_j(\mu\partial_j u_i) + \partial_i(\mu\partial_k u_i) + c_0\partial_k(\mu\partial_i u_i) = f_k. \quad \text{(Supplementary Equation 171)}$$

To determine the probe intensity, we expand the deformation field to leading order in $\delta\lambda(\boldsymbol{r})$. This approach yields an approximate deformation field given by the sum of a zeroth order deformation field $u_i^{(0)}$ and a first order deformation field $u_i^{(1)}$. We solve for the zeroth order integrated measurement $m^{(0)}$ by inverting the above constitutive relation to find:

$$u_i^{(0)} = \frac{1}{\mu_0} \int G_{ik} f_k d\boldsymbol{r} \quad \text{(Supplementary Equation 172)}$$

where G_{ik} is the response function defined by:

$$(\delta_{i,k}\partial_j\partial_j + \partial_i\partial_k + c_0\partial_i\partial_k)G_{ia} = \delta_{k,a}. \quad \text{(Supplementary Equation 173)}$$

The leading order integrated measurement $m^{(0)}$ is given by:

$$m^{(0)} = \int w_i u_i^{(0)} d\boldsymbol{r}. \quad \text{(Supplementary Equation 174)}$$

$$m^{(0)} = \frac{1}{\mu_0} \int w_i V_{f,i} d\boldsymbol{r}, \quad \text{(Supplementary Equation 175)}$$

where $V_{f,i}$ is the stimulus potential:

$$V_{f,i} = \int G_{ik} f_k d\boldsymbol{r}. \qquad \text{(Supplementary Equation 176)}$$

Equivalently, the stimulus potential is also defined by the following equation:

$$(\delta_{i,k}\partial_j\partial_j + \partial_i\partial_k + c_0\partial_k\partial_i)V_{f,i} = f_k. \qquad \text{(Supplementary Equation 177)}$$

Similarly, we define a weight potential:

$$V_{w,i} = \int G_{ik} w_k d\boldsymbol{r}. \qquad \text{(Supplementary Equation 178)}$$

$$(\delta_{i,k}\partial_j\partial_j + \partial_i\partial_k + c_0\partial_k\partial_i)V_{w,i} = w_k. \qquad \text{(Supplementary Equation 179)}$$

Inserting Supplementary Equation 179 into Supplementary Equation 175 yields:

$$m^{(0)} = \frac{1}{\mu_0}\int(\delta_{i,k}\partial_j\partial_j + \partial_k\partial_i + c_0\partial_i\partial_k)V_{w,k}V_{f,i}d\boldsymbol{r}. \qquad \text{(Supplementary Equation 180)}$$

We integrate by parts to find:

$$m^{(0)} = \frac{1}{\mu_0}\int(\partial_j V_{f,i}\partial_j V_{w,i} + \partial_i V_{f,k}\partial_k V_{w,i} + c_0\partial_i V_{f,i}\partial_k V_{w,k})d\boldsymbol{r}. \qquad \text{(Supplementary Equation 181)}$$

We now turn to the first order integrated measurement $m^{(1)}$. To leading order in $\delta\mu$, the first-order deformation field $u_i^{(1)}$ is:

$$u_i^{(1)} = \frac{-1}{\mu_0^2}\int G_{ik}\left(\delta_{i,k}\partial_j(\delta\mu\partial_j V_{f,i}) + \partial_i(\delta\mu\partial_k V_{f,i}) + c_0\partial_k(\delta\mu\partial_i V_{f,i})\right)d\boldsymbol{r}. \qquad \text{(Supplementary Equation 182)}$$

Thus, the first-order integrated measurement $m^{(1)}$ is:

$$m^{(1)} = \int w_i u_i^{(1)} d\boldsymbol{r} = \frac{-1}{\mu_0^2}\int\left(V_{w,i}\partial_j(\delta\mu\partial_j V_{f,i}) + V_{w,k}\partial_i(\delta\mu\partial_k V_{f,i}) + c_0 V_{w,k}\partial_k(\delta\mu\partial_i V_{f,i})\right)d\boldsymbol{r}.$$

(Supplementary Equation 183)

We integrate by parts to find:

$$m^{(1)} = \frac{-1}{\mu_0^2}\int\delta\mu\left(\partial_j V_{w,i}\partial_j V_{f,i} + \partial_i V_{w,k}\partial_k V_{f,i} + c_0\partial_k V_{w,k}\partial_i V_{f,i}\right)d\boldsymbol{r}. \qquad \text{(Supplementary Equation 184)}$$

Adding together Supplementary Equation 181 and Supplementary Equation 184 results in the following integrated measurement $m \equiv m^{(0)} + m^{(1)}$:

$$m = \int\left(\frac{1}{\mu_0} - \frac{\delta\mu(\boldsymbol{r})}{\mu_0^2}\right)\psi(\boldsymbol{r})d\boldsymbol{r}, \qquad \text{(Supplementary Equation 185)}$$

where we have defined the probe intensity $\psi(\boldsymbol{r})$:

$$\psi(\boldsymbol{r}) = \partial_j V_{f,i}\partial_j V_{w,i} + \partial_i V_{f,k}\partial_k V_{w,i} + c_0\partial_i V_{f,i}\partial_k V_{w,k}. \qquad \text{(Supplementary Equation 186)}$$

By analogy to the elastic sheet, it follows that the sensor can obtain an unbiased estimate of μ_0 for the elastic medium by inserting this probe intensity into equation (7).

(ii) Estimating the material parameters for a biopolymer network.

To model cellular mechanosensing, we chose the parameters of the elastic medium to describe a reconstituted collagen network[8–11]. Previous studies have measured the bulk response of reconstituted collagen networks[12–15]. However, to our knowledge, no studies have reported the parameters Δ_μ and ξ used in our model to characterize the local heterogeneity. To determine these parameters, we fit our continuum model to the results of Ref. 11 as follows:

- The shear modulus μ_0 was taken to be the value $\mu_0 \simeq 0.3\,\mathrm{Pa}$ measured for the experimental network in Ref. 11 via bulk rheology.
- The Poisson's ratio σ was not reported in Ref. 11. Thus, we take its value to be $\sigma \simeq 0.4$, consistent with other previous studies of collagen networks[14,16].
- To determine Δ_μ, we fit our continuum model to the local stiffness distribution obtained for the simulated response of the discrete collagen network in Ref. 11. To compare our sensing model to the discrete force dipole used in Ref. 11, we took the probe in our sensing model to be a completely anisotropic force dipole. The deformation field produced by such a dipole is proportional to:

  $$V_i(\boldsymbol{r}) = G_{ij,k}(\boldsymbol{r})P_{jk}, \qquad \text{(Supplementary Equation 187)}$$

  where $G_{ij,k}(\boldsymbol{r})$ is the gradient of the response function $G_{ij}(\boldsymbol{r})$ for a continuous elastic medium[17]:

  $$G_{ij}(\boldsymbol{r}) = \left[(3-4\sigma)\delta_{i,j} + \hat{r}_i\hat{r}_j\right]\frac{1}{r}. \qquad \text{(Supplementary Equation 188)}$$

  In this expression, $\hat{\boldsymbol{r}}$ is a unit vector oriented along $\boldsymbol{r}$, and P_{jk} is the dipole moment tensor[18]. For a completely anisotropic dipole, this tensor can be expressed as:

  $$P_{jk} = \delta_{1,j}\delta_{1,k}. \qquad \text{(Supplementary Equation 189)}$$

  In contrast to the force dipole applied to the discrete network in Ref. 11, a force dipole in the continuum limit induces diverging deformations at the points where the forces are applied. To account for these unphysical divergences, we take the measurement protocol to include a spherical cutoff region of radius a equal to the length of the dipoles in Ref. 11. Applying this cutoff to the deformation field in Supplementary Equation 187 results in the following probe potentials:

  $$V_{f,i}(\boldsymbol{r}) \sim V_{w,i}(\boldsymbol{r}) \sim \begin{cases} \partial_k\left(\left[(3-4\sigma)\delta_{i,j} + \hat{r}_i\hat{r}_j\right]\frac{r^2}{a^3}\right)P_{jk}, & r < a, \\ \partial_k\left(\left[(3-4\sigma)\delta_{i,j} + \hat{r}_i\hat{r}_j\right]\frac{1}{r}\right)P_{jk}, & r > a. \end{cases} \qquad \text{(Supplementary Equation 190)}$$

  Using this measurement protocol, we determined Δ_μ by computing the fractional uncertainty Δ_μ/μ_0 via numerical integration and setting it equal to the corresponding fractional uncertainty found for the local response in Ref. 11, i.e. the standard deviation of the local stiffness distribution divided by its mean. This comparison resulted in a value of $\Delta_\mu \sim 0.1\,\mathrm{Pa}^2$.
- The correlation length ξ of the fluctuations in the material constant was determined by fitting the covariance of two continuum dipoles of a given separation (calculated using (Supplementary Equation 97) to the covariance measured for two network dipoles in Ref. 11. This fit yielded a value $\xi \sim 5\,\mu\mathrm{m}$.

(iii) The cellular probe of stiffness.

In mechanical equilibrium, a cell cannot exert a net force on the medium due to the requirement of force balance. Under this restriction, a cell in $D = 3$ maximizes its effective range by applying probe potentials that decay as $\sim 1/r^2$ in the far-field limit. The simplest possible measurement protocol with such a profile consists of isotropic dipolar shells of radius a:

$$f_i(\boldsymbol{r}) \sim \delta(r-a)\hat{\boldsymbol{r}}, \qquad \text{(Supplementary Equation 191)}$$

$$w_i(\boldsymbol{r}) \sim \delta(r-a)\hat{\boldsymbol{r}}. \qquad \text{(Supplementary Equation 192)}$$

These probe vector fields produce the following probe intensity:

$$\psi(\boldsymbol{r}) \sim \begin{cases} 1+3\varsigma/2, & r<a. \\ 2r^{-6}, & r>a. \end{cases} \qquad \text{(Supplementary Equation 193)}$$

We insert this probe intensity into Supplementary Equation 31 to obtain the variance $\delta\mu_0^2$ in the cell's estimate of μ_0:

$$\delta\mu_0^2 = \Delta_\mu \xi^D V^{-1}\left(\frac{27\varsigma^2+36\varsigma+28}{27\varsigma^2+108\varsigma+108}\right). \qquad \text{(Supplementary Equation 194)}$$

Supplementary Note 12 Performing multiple probes in different locations

In previous sections, we focused on a localized sensor with stimulus and weight fields f and w nonzero only within a sphere of radius a. We have shown that such sensors can greatly improve the precision of their estimates of material properties by multiplexing measurements with different multipole symmetries. In this section, we consider an alternative approach for estimating material properties, in which the sensor always exerts a probe with the same symmetry, but moves a fixed distance between probes. Such a strategy could be adopted by a motile cell or microrobot. Strikingly, we find that for the same total number N of measurements, a sensor that remains in one place and applies different multipole probes generically does better than a moving sensor. Specifically, the fractional error drops off like $1/\sqrt{N}$ for the moving sensor but faster than $1/\sqrt{N}$ for the stationary sensor. Nevertheless, a stationary sensor is limited by its resolution d. Thus, when a material property must be estimated in a limited amount of time, it may often be preferable for the sensor to stay put, but when time is not an important constraint, a moving sensor will eventually do better.

We begin by considering our analytic results for scalar elasticity to gain insight into how the bound on precision scales with N. For the two-dimensional elastic sheet, $\delta\lambda_0^2 \sim 1/(k_{\max}+1)^2$ (see Sensory multiplexing can significantly improve the precision of sensing). The number N of distinct probes for each k does not depend on k, so that $N \sim k_{\max}$ and $\delta\lambda_0^2 \sim 1/N^2$. For the three-dimensional case, from Supplementary Equation 150 we find that $\delta\lambda_0^2 \sim 1/\ell_{\max}^3$. The number of distinct probes for each ℓ goes like ℓ, so that $N \sim \ell_{\max}^2$, and $\delta\lambda_0^2 \sim 1/N^{3/2}$.

We next turn to the case of a moving sensor. For concreteness, we consider a sensor moving in a straight line along the x axis and exerting a dipolar probe each time it has gone a distance $2a$. We focus on scalar elasticity in two dimensions and vectorial elasticity in three dimensions. For the former, the probe intensity of probe j is, from equation (19) with $\gamma = 1$,

$$\psi_j(\boldsymbol{r}) \sim \begin{cases} 1, & |\boldsymbol{r}-2ja\hat{\boldsymbol{x}}|< a. \\ a^4|\boldsymbol{r}-2ja\hat{\boldsymbol{x}}|^{-4}, & |\boldsymbol{r}-2ja\hat{\boldsymbol{x}}|> a. \end{cases} \qquad \text{(Supplementary Equation 195)}$$

Similarly, the probe fields for isotropic dipolar probes performed at positions $2ja\hat{\boldsymbol{x}}$ in a three-dimensional elastic medium are

$$f_i(\boldsymbol{r}) \sim \delta(|\boldsymbol{r}-2ja\hat{\boldsymbol{x}}|-a)\frac{\boldsymbol{r}-2ja\hat{\boldsymbol{x}}}{|\boldsymbol{r}-2ja\hat{\boldsymbol{x}}|}, \qquad \text{(Supplementary Equation 196)}$$

$$w_i(\boldsymbol{r}) \sim \delta(|\boldsymbol{r}-2ja\hat{\boldsymbol{x}}|-a)\frac{\boldsymbol{r}-2ja\hat{\boldsymbol{x}}}{|\boldsymbol{r}-2ja\hat{\boldsymbol{x}}|}, \qquad \text{(Supplementary Equation 197)}$$

where $\hat{\boldsymbol{x}}$ is a unit vector specifying the direction of the cell's motion. These probe vector fields produce the following probe intensities:

$$\psi_j(\boldsymbol{r}-2ja\hat{\boldsymbol{x}}) \sim \begin{cases} 1+3\varsigma/2, & |\boldsymbol{r}-2ja\hat{\boldsymbol{x}}|< a. \\ 2a^6|\boldsymbol{r}-2ja\hat{\boldsymbol{x}}|^{-6}, & |\boldsymbol{r}-2ja\hat{\boldsymbol{x}}|> a. \end{cases} \qquad \text{(Supplementary Equation 198)}$$

The variance $\delta\lambda_0^2$ in the sensor's estimate of λ_0 is given by:

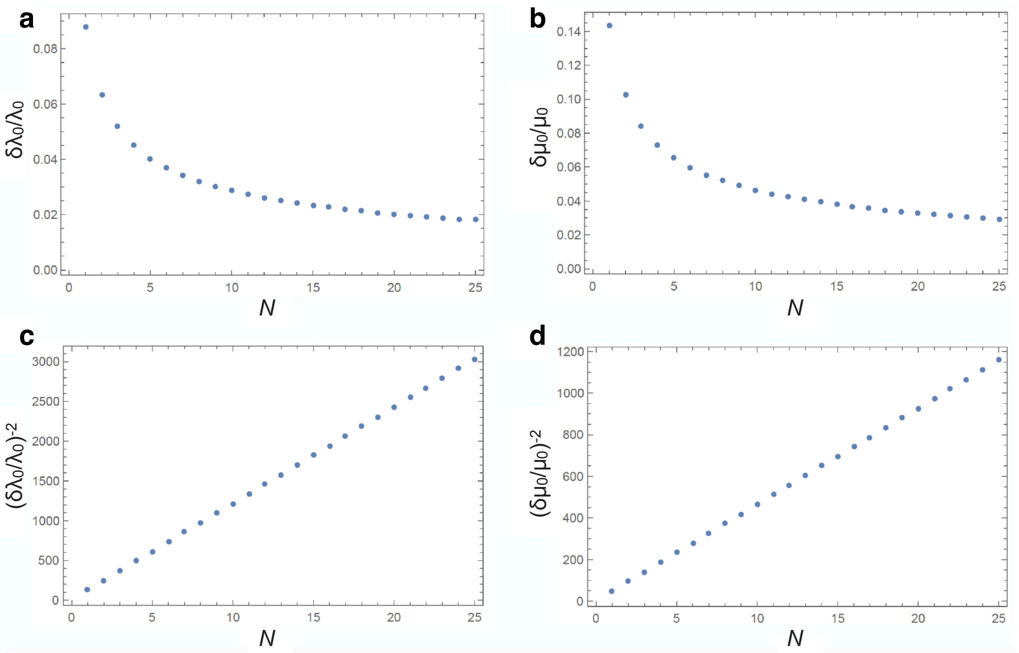

Supplementary Figure 1. Performing multiple probes in different locations can improve the precision of sensing (**a,b**) Fractional uncertainties (**a**) $\delta\lambda_0/\lambda_0$ and (**b**) $\delta\mu_0/\mu_0$ for a sensor of radius a that travels in a straight line and performs a probe at every distance $2a$ versus the number N of distinct measurements performed for (**a**) a two-dimensional elastic sheet and (**b**) the three-dimensional collagen network considered in the main text (see The precision of biomechanical sensing). (**c,d**) Squared inverse fractional uncertainties (**c**) $(\lambda_0/\delta\lambda_0)^2$ and (**d**) $(\mu_0/\delta\mu_0)^2$ for a sensor of radius a that travels in a straight line and performs a probe at every distance $2a$ versus the number N of distinct measurements performed for (**c**) a two-dimensional elastic sheet and (**d**) the three-dimensional collagen network considered in the main text (see The precision of biomechanical sensing). For elastic sheet in (**a,c**), $\sqrt{\Delta_\lambda}/\lambda_0 = 0.25$ and $\xi/a = 0.5$.

$$\delta\lambda_0^2 = \left(\sum_{k,l} C_{kl}^{-1}\right)^{-1}, \quad \text{(Supplementary Equation 199)}$$

where

$$C_{kl} = \langle(\hat{\lambda}_{0,k} - \lambda_0)(\hat{\lambda}_{0,l} - \lambda_0)\rangle \sim \int \psi_i(\boldsymbol{r})\psi_j(\boldsymbol{r})d^D\boldsymbol{r} \quad \text{(Supplementary Equation 200)}$$

is the covariance matrix of the estimators for the individual probes. Analogous equations hold for $\delta\mu/\mu_0$ in the case of vectorial elasticity. Supplementary Fig. 1 shows that $1/\delta\lambda_0^2$ and $1/\delta\mu_0^2$ both scale linearly with N.

How can this scaling be understood? First note that if different probes were completely uncorrelated, i.e. $C_{kl} = 0$ for $k \neq l$, then the covariance matrix would be proportional to the identity matrix, and we would expect $\delta\lambda_0^2 \sim 1/N$. We then observe that from our expressions for ψ_j, C_{kl} decays as $1/|k-l|^4$ in the two-dimensional case and $1/|k-l|^6$ in the three-dimensional case. This rapid decay suggests that correlations between probes may be weak enough that the uncorrelated $1/N$ scaling is recovered for large N. To be concrete, let $v_j = C_{jk}^{-1} 1_k$, where 1_k is the kth component of a vector with all entries equal to 1. Then $C_{kj}v_j = 1_k$. Using the fact that C_{jk} depends only on $|j-k|$, we define $C_{|j-k|} \equiv C_{jk}$. For k far from 1 and N — in other words, ignoring "boundary effects" — the above equation reduces to

$$... + C_2 v_{k-2} + C_1 v_{k-1} + C_0 v_k + C_1 v_{k+1} + C_2 v_{k+2} + ... = 1, \quad \text{(Supplementary Equation 201)}$$

for each k. As C_n drops off fast enough with n that the sum over n converges, Supplementary Equation 201 is solved in the limit of large N by taking all components v_k to have the same value

$$v_k \approx \left(C_0 + 2\sum_{n=1}^{\infty} C_n \right)^{-1} 1_k. \qquad \text{(Supplementary Equation 202)}$$

Then,

$$1/\delta\lambda_0^2 = 1_j C_{jk}^{-1} 1_k = 1_j v_j \sim 1_j^2 = N. \qquad \text{(Supplementary Equation 203)}$$

Thus, we conclude that in the large N limit, the precision bound for a moving sensor decreases less rapidly with N than the scaling we found for a stationary sensor.

Supplementary Note 13 One-particle active microrheology

In this Supplementary Note, we apply our theoretical framework to estimate the precision of a stiffness probe that consists of a single bead of radius a embedded inside an elastic medium. We model the elastic medium as before in Supplementary Note 11. To model a probe of stiffness that consists of a single bead, we take the stimulus and weight fields to be monopoles oriented in the same direction f_i. The stimulus and weight fields produced by such a probe are proportional to:

$$V_{f,i}(\boldsymbol{r}) \sim V_{w,i}(\boldsymbol{r}) \sim G_{ij}(\boldsymbol{r}) f_j, \qquad \text{(Supplementary Equation 204)}$$

where $G_{ij}(\boldsymbol{r})$ is the response function for a continuous elastic medium given by Supplementary Equation 188. These probe fields diverge at the point where they are applied. To account for these unphysical divergences, we take the measurement protocol to include a spherical cutoff region of radius ξ. Applying this cutoff to the deformation field in Supplementary Equation 204 results in the following probe potentials:

$$V_{f,i}(\boldsymbol{r}) \sim V_{w,i}(\boldsymbol{r}) \sim \left([(3-4\sigma)\delta_{i,j} + \hat{r}_i \hat{r}_j] \frac{1}{r} \right) f_j \delta(r-\xi) \qquad \text{(Supplementary Equation 205)}$$

In contrast to the cellular probe of stiffness we considered in the main text, this measurement protocol breaks radial symmetry. Thus, it is possible to extract additional information by performing multiple probes in different directions and combining the results via sensory multiplexing. To quantify the effectiveness of this strategy, we calculated the fractional uncertainty for a series of probes performed by a single bead at a fixed equilibrium position under probe fields applied in different directions. Intuitively, a fixed number of probes can obtain the largest amount of information by maximally spreading out the directions they probe. For simplicity, we considered a series of sets of directions given by the following minimization:

$$\underset{\boldsymbol{f}_i}{\text{minimize}} \quad \sum_{i \neq j} (\boldsymbol{f}_i \cdot \boldsymbol{f}_j)^2 \qquad \text{(Supplementary Equation 206)}$$

We performed this minimization using simulated annealing, and then used the resulting measurement protocols to determine the fractional uncertainty Δ_μ/μ_0 for the collagen network in Supplementary Note 11 as a function of the number of probes N contained in each measurement protocol. To that end, we evaluated Supplementary Equation 199 via numerical integration. This procedure resulted in values of $\delta\mu_0/\mu_0$ that decrease with N, but with negligible improvements beyond $N = 3$ (see Supplementary Fig. 2).

Supplementary Note 14 Probing a Winkler foundation with a finite correlation length

In the main text, we focused on the limit $\xi \ll d$. However, our theoretical framework also applies when ξ is comparable in size to the sensor. In this Supplementary Note, we revisit sensing without assuming $\xi \ll d$. To simplify our analysis, we take the medium to be the simplest heterogeneous material: a disordered Winkler foundation[19]. This

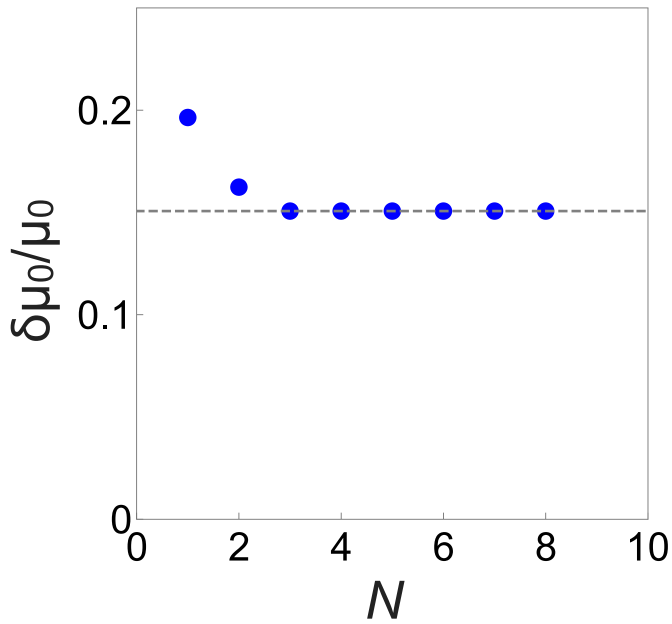

Supplementary Figure 2. Performing multiple probes in different directions can improve the precision of microrheology. Fractional uncertainties $\delta\mu_0/\mu_0$ for measurement protocols that consist of N monopoles performed in different directions (see Supplementary Note 12). Dashed line shows fractional uncertainty for $N = 8$ probes.

medium corresponds to an array of decoupled springs in the continuum limit. The internal energy of the Winkler foundation is given by:

$$E = \frac{1}{2}\int \lambda(\boldsymbol{r})u(\boldsymbol{r})^2 d\boldsymbol{r}. \qquad \text{(Supplementary Equation 207)}$$

Here, as in the main text, we take $\lambda(\boldsymbol{r})$ to be a Gaussian random field with mean λ_0, variance $\Delta_\lambda \ll \lambda_0^2$, and spatial correlations over a scale ξ. As before, we take the sensor to interact with the medium within a radius a by first applying a stimulus field $f(\boldsymbol{r})$ as in equation (4), and then measuring an integrated response m as in equation (5).

To leading order in $\delta\lambda(\boldsymbol{r})$, the sensor can again compute $\hat{\lambda}_0 = s/m$ to obtain a spatial average of $\lambda(\boldsymbol{r})$ weighted by a probe intensity $\psi(\boldsymbol{r})$, as in equation (9). However, for the Winkler foundation, $\psi(\boldsymbol{r})$ is now:

$$\psi(\boldsymbol{r}) = f(\boldsymbol{r})w(\boldsymbol{r}). \qquad \text{(Supplementary Equation 208)}$$

By analogy to the elastic sheet, the sensor again obtains an unbiased estimate of λ_0 using equation (7). In this case, however, the variance $\delta\lambda_0^2$ is given by:

$$\delta\lambda_0^2 = \frac{\int\int\langle\delta\lambda(\boldsymbol{r}_1)\delta\lambda(\boldsymbol{r}_2)\rangle\psi(\boldsymbol{r}_1)\psi(\boldsymbol{r}_2)d\boldsymbol{r}_1 d\boldsymbol{r}_2}{\left(\int\psi(\boldsymbol{r})d\boldsymbol{r}\right)^2}. \qquad \text{(Supplementary Equation 209)}$$

For the short-ranged correlations described by equation (3), this variance reduces to Supplementary Equation 31. Moreover, the variance is invariant with respect to an overall rescaling of $\psi(\boldsymbol{r})$. To eliminate this redundancy, we constrain $\int\psi(\boldsymbol{r})d\boldsymbol{r}$ to be a fixed constant. Furthermore, we must enforce $\psi(\boldsymbol{r}) = 0$ in the exterior of the sensor ($r > a$) to satisfy the constraints imposed by the finite size of the sensor. Thus, the minimum of Supplementary Equation 31 is determined by the configuration of $\psi(\boldsymbol{r})$ that minimizes the following action S:

$$S = \int_{\mathcal{R}_{\text{int}}}\left(\frac{1}{2}\psi(\boldsymbol{r})^2 - \gamma\psi(\boldsymbol{r})\right)d\boldsymbol{r}, \qquad \text{(Supplementary Equation 210)}$$

where the integral is taken over the interior $\mathcal{R}_{\text{int}}$ of the sensor $(r < a)$ and γ is a Lagrange multiplier that fixes $\int \psi(\boldsymbol{r}) d\boldsymbol{r}$. This action is minimized by any $\psi(\boldsymbol{r})$ that is uniform over $\mathcal{R}_{\text{int}}$, e.g. $f(\boldsymbol{r}) \sim w(\boldsymbol{r}) \sim 1$. The optimal measurement protocol for $\xi \ll d$ is therefore:

$$\psi(\boldsymbol{r}) = \begin{cases} \gamma, & r < a. \\ 0, & r > a. \end{cases} \qquad \text{(Supplementary Equation 211)}$$

This $\psi(\boldsymbol{r})$ exhaustively samples the information that can be gleaned by interacting with the Winkler foundation in the interior $(r < a)$, even for a sensor that can perform multiple measurements. This fact follows from considering a convex relaxation of the effective probe intensity $\Psi(\boldsymbol{r})$ for multiple measurements that consists of allowing $\Psi(\boldsymbol{r})$ to take on arbitrary configurations in the interior of the sensor and fixed to $\Psi(\boldsymbol{r}) = 0$ in the exterior $(r > a)$. The optimal effective probe intensity for this convex relaxation is $\Psi(\boldsymbol{r}) \sim \gamma$ for $r < a$, which coincides with Supplementary Equation 211. Inserting Supplementary Equation 211 into Supplementary Equation 31 yields:

$$\delta\lambda_0^2 = \Delta_\lambda \xi^D V^{-1}, \qquad \text{(Supplementary Equation 212)}$$

where V is the volume of the sensor. Thus, the fractional uncertainty of the estimator $\hat{\lambda}_0$, defined as the standard deviation $\delta\lambda_0$ divided by the mean λ_0, scales as:

$$\frac{\delta\lambda_0}{\lambda_0} \sim \left(\frac{\Delta_\lambda}{\lambda_0^2}\right)^{1/2} \left(\frac{\xi}{a}\right)^{D/2}, \qquad \text{(Supplementary Equation 213)}$$

for $\xi \ll d$. To see how these results would change if we do not assume $\xi \ll d$, we instead take the correlations to be described by:

$$\langle \delta\lambda(\boldsymbol{r}_1) \delta\lambda(\boldsymbol{r}_2) \rangle = \Delta_\lambda e^{-|\boldsymbol{r}_1 - \boldsymbol{r}_2|/\xi}. \qquad \text{(Supplementary Equation 214)}$$

Inserting Supplementary Equation 211 and Supplementary Equation 214 into Supplementary Equation 209 gives:

$$\delta\lambda_0^2 = \Delta_\lambda V^{-2} \int\int e^{-|\boldsymbol{r}_1 - \boldsymbol{r}_2|/\xi} d\boldsymbol{r}_1 d\boldsymbol{r}_2. \qquad \text{(Supplementary Equation 215)}$$

For $D = 3$, we compute these integrals by switching to spherical coordinates to find:

$$\delta\lambda_0^2 = \frac{3\Delta_\lambda \xi^3}{16\pi a^6} \left(4a^3 - 9a^2\xi + 15\xi^3 - 3e^{-2a/\xi}(a+\xi)(2a^2 + 5a\xi + 5\xi^2)\right). \qquad \text{(Supplementary Equation 216)}$$

This variance increases with ξ and saturates to a constant value $\Delta_\lambda/(8\pi)$ in the limit $\xi \gg a$. Thus, in this regime, the fractional uncertainty for the Winkler foundation is proportional to the local noise-to-signal ratio $\Delta_\lambda^{1/2}/\lambda_0$ of the material constant field. For elastic media, we expect this scaling to apply when the correlation volume is much larger than the effective volume sampled by the sensor.

Supplementary References

[1] E. L. Lehmann and H. Scheffé, Completeness, similar regions, and unbiased estimation-part i, in *Selected Works of E. L. Lehmann* (Springer US, 2011) pp. 233–268.

[2] E. L. Lehmann and H. Scheffé, Completeness, similar regions, and unbiased estimation—part II, in *Selected Works of E. L. Lehmann* (Springer US, 2011) pp. 269–286.

[3] G. A. Young and R. L. Smith, Sufficiency and completeness, in *Essentials of Statistical Inference* (Cambridge University Press) pp. 90–97.

[4] R. A. Fisher, On the mathematical foundations of theoretical statistics, Philosophical Transactions of the Royal Society A: Mathematical, Physical and Engineering Sciences **222**, 309 (1922).

[5] R. Bellman, *Laplace Transform, The (Series in Modern Applied Mathematics)* (Wspc, 1984).

[6] P. Chareka, A finite-interval uniqueness theorem for bilateral laplace transforms, International Journal of Mathematics and Mathematical Sciences **2007**, 1 (2007).
[7] J. D. Jackson, *Classical Electrodynamics* (John Wiley & Sons Inc, 1998).
[8] A. D. Doyle and K. M. Yamada, Mechanosensing via cell-matrix adhesions in 3d microenvironments, Experimental Cell Research **343**, 60 (2016).
[9] M. H. Zaman, L. M. Trapani, A. L. Sieminski, D. MacKellar, H. Gong, R. D. Kamm, A. Wells, D. A. Lauffenburger, and P. Matsudaira, Migration of tumor cells in 3d matrices is governed by matrix stiffness along with cell-matrix adhesion and proteolysis, Proceedings of the National Academy of Sciences **103**, 10889 (2006).
[10] Q. Guo, J. M. Phillip, S. Majumdar, P.-H. Wu, J. Chen, X. Calderón-Colón, O. Schein, B. J. Smith, M. M. Trexler, D. Wirtz, and J. H. Elisseeff, Modulation of keratocyte phenotype by collagen fibril nanoarchitecture in membranes for corneal repair, Biomaterials **34**, 9365 (2013).
[11] F. Beroz, L. M. Jawerth, S. Münster, D. A. Weitz, C. P. Broedersz, and N. S. Wingreen, Physical limits to biomechanical sensing in disordered fibre networks, Nature Communications **8**, 16096 (2017).
[12] P. A. Janmey, E. J. Amis, and J. D. Ferry, Rheology of fibrin clots. VI. stress relaxation, creep, and differential dynamic modulus of fine clots in large shearing deformations, Journal of Rheology **27**, 135 (1983).
[13] B. A. Roeder, K. Kokini, J. E. Sturgis, J. P. Robinson, and S. L. Voytik-Harbin, Tensile mechanical properties of three-dimensional type i collagen extracellular matrices with varied microstructure, Journal of Biomechanical Engineering **124**, 214 (2002).
[14] D. M. Knapp, V. H. Barocas, A. G. Moon, K. Yoo, L. R. Petzold, and R. T. Tranquillo, Rheology of reconstituted type i collagen gel in confined compression, Journal of Rheology **41**, 971 (1997).
[15] R. C. Arevalo, J. S. Urbach, and D. L. Blair, Size-dependent rheology of type-i collagen networks, Biophysical Journal **99**, L65 (2010).
[16] A. P. G. Castro, P. Laity, M. Shariatzadeh, C. Wittkowske, C. Holland, and D. Lacroix, Combined numerical and experimental biomechanical characterization of soft collagen hydrogel substrate, Journal of Materials Science: Materials in Medicine **27**, 10.1007/s10856-016-5688-3 (2016).
[17] A. M. . P. L. L. D. Landau; Lifshitz, E. M. ; Kosevich, *Theory of Elasticity* (Pergamon Press, 1986).
[18] T. J. Emmanuel Clouet, Celine Varvenne, Elastic modeling of point-defects and their interaction, Computational Materials Science (2018).
[19] E. Winkler, *Die Lehre von der Elasticitaet und Festigkeit: mit besonderer Rücksicht auf ihre Anwendung in der Technik, für polytechnische Schulen, Bauakademien, Ingenieure, Maschinenbauer, Architecten, etc*, Die Lehre von der Elastizität und Festigkeit mit besonderer Rücksicht auf ihre Anwendung in der Technik: für polytechnische Schulen, Bauakademien, Ingenieure, Maschinenbauer, Architecten, etc (Dominicius, 1868).
